# Interrogation of the *Burkholderia pseudomallei* Genome to Address Differential Virulence among Isolates

**DOI:** 10.1371/journal.pone.0115951

**Published:** 2014-12-23

**Authors:** Jean F. Challacombe, Chris J. Stubben, Christopher P. Klimko, Susan L. Welkos, Steven J. Kern, Joel A. Bozue, Patricia L. Worsham, Christopher K. Cote, Daniel N. Wolfe

**Affiliations:** 1 Los Alamos National Laboratory, Bioscience Division, Los Alamos, NM, United States of America; 2 US Army Medical Research Institute of Infectious Diseases, Bacteriology Division, Fort Detrick, MD, United States of America; 3 US Army Medical Research Institute of Infectious Diseases, Biostatistics Division, Fort Detrick, MD, United States of America; 4 Defense Threat Reduction Agency, Chemical and Biological Technologies Department, Fort Belvoir, VA, United States of America; J. Craig Venter Institute, United States of America

## Abstract

Infection by the Gram-negative pathogen *Burkholderia pseudomallei* results in the disease melioidosis, acquired from the environment in parts of southeast Asia and northern Australia. Clinical symptoms of melioidosis range from acute (fever, pneumonia, septicemia, and localized infection) to chronic (abscesses in various organs and tissues, most commonly occurring in the lungs, liver, spleen, kidney, prostate and skeletal muscle), and persistent infections in humans are difficult to cure. Understanding the basic biology and genomics of *B. pseudomallei* is imperative for the development of new vaccines and therapeutic interventions. This formidable task is becoming more tractable due to the increasing number of *B. pseudomallei* genomes that are being sequenced and compared.

Here, we compared three *B. pseudomallei* genomes, from strains MSHR668, K96243 and 1106a, to identify features that might explain why MSHR668 is more virulent than K96243 and 1106a in a mouse model of *B. pseudomallei* infection. Our analyses focused on metabolic, virulence and regulatory genes that were present in MSHR668 but absent from both K96243 and 1106a. We also noted features present in K96243 and 1106a but absent from MSHR668, and identified genomic differences that may contribute to variations in virulence noted among the three *B. pseudomallei* isolates. While this work contributes to our understanding of *B. pseudomallei* genomics, more detailed experiments are necessary to characterize the relevance of specific genomic features to *B. pseudomallei* metabolism and virulence. Functional analyses of metabolic networks, virulence and regulation shows promise for examining the effects of *B. pseudomallei* on host cell metabolism and will lay a foundation for future prediction of the virulence of emerging strains. Continued emphasis in this area will be critical for protection against melioidosis, as a better understanding of what constitutes a fully virulent *Burkholderia* isolate may provide for better diagnostic and medical countermeasure strategies.

## Introduction

Melioidosis, the disease caused by *Burkholderia pseudomallei*, presents with a wide range of non-specific signs and symptoms, including fever, pneumonia, acute septicemia, and chronic localized infection [1]–[3]. Initial infection can also be asymptomatic. Chronic stages of the disease are characterized by abscesses in various organs and tissues, most commonly occurring in the lungs, liver, spleen, kidney, prostate and skeletal muscle [1], [3], [4]. Melioidosis is community-acquired through bacterial contamination of wounds, inhalation, and ingestion [5]. Research in Thailand and Australia has provided critical information about the clinical epidemiology of the disease; the clinical presentations of melioidosis caused by Thai and Australian strains differ in several ways: 1) parotid abscesses are not prevalent in Australia, but occur in Thailand; 2) prostate abscesses are uncommon in Thailand, but are more commonly seen in Australia [3]; and 3) an encephalomyelitis syndrome is seen in tropical Australia more often than in Thailand [5]. This latter condition was associated with the illnesses caused by *B. pseudomallei* strains MSHR668 [6] and MSHR305 [7]. However, there is evidence that the same strain can cause different clinical presentations in different individuals, and a number of risk factors, such as diabetes have been identified for melioidosis [8]. Therefore, host factors may be important in determining the severity and duration of disease [9], [10].

The high incidences of infection in geographical areas where *B. pseudomallei* is endemic may be due to its resilience and ability to survive under sometimes harsh environmental conditions. *B. pseudomallei* can survive nutrient depletion, a wide range of pH differences, salt concentrations, and temperatures [11], detergent solutions [12] and acidic environments [13]. It seems that harsh environmental conditions may confer a selective advantage for the growth of *B. pseudomallei* [5]. These resilience characteristics may explain why *B. pseudomallei* can cause persistent infections in the human host that are difficult to cure. Also, *B. pseudomallei* is naturally resistant to a variety of antimicrobial agents [14], [15]. In some cases, there is a latency period before symptoms present that can last for days to years [5]. In other cases, an initial acute infection and extensive antibiotic treatment is followed by a variable period of bacterial persistence, with subsequent recrudescence of the disease months or years after the initial infection [16], [17].

Our understanding of *B. pseudomallei* pathogenesis is further complicated by the natural diversity of its genome. *B. pseudomallei* is a soil-dwelling bacterium that utilizes lateral gene transfer at a very high rate [18]. As a result, there is substantial variation among *B. pseudomallei* genomes, which may also contribute to differential virulence. Fortunately, as we now have access to many *B. pseudomallei* genomes from various geographic locations, it is possible to identify genomic features that the various strains have in common, as well as features that are unique to one or more strains.

Comparative studies of genomes from Australian and Thai *B. pseudomallei* isolates have revealed genomic differences that contribute to our understanding of this organism. The genomes of *B. pseudomallei* analyzed so far contain from 16–21 genomic islands (GIs) [7], [19], [20]. The genome of *B. pseudomallei* strain K96243 contains 16 GIs [19] that are variably present in other *B. pseudomallei* genomes [20], and each GI shows micro-evolutionary changes that generate GI diversity [20]. In addition to GIs, the genomes of Thai strains K96243 and 1106a contain a horizontally acquired Yersinia-like fimbrial (YLF) gene cluster, while the comparable region in the Australian strains (MSHR668, MSHR305, DM98, 1655 and 13177) is the *B. thailandensis*-like flagellum and chemotaxis (BTFC) gene cluster [21]. Previous studies showed that BTFC is dominant in Australian strains, while YLF is dominant in strains from Thailand and elsewhere [21]. In addition, clinical isolates are more likely to belong to group YLF, whereas environmental isolates are more likely to belong to group BTFC [21]. In contrast to these trends, we found that the Australian strain MSHR346 contains the YLF cluster (data not shown), and Tuanyok and colleagues reported that 406e, a clinical isolate from Thailand, has BTFC [21].

Previous studies began to address the question of why different strains show differences in virulence and disease presentation. Many studies have focused on host risk factors such as diabetes and alcoholism; but to date only one study has identified genes associated with different disease presentations [22]. This suggests that virulence factors that are variably present in *B. pseudomallei* strains may be important for pathogenesis. Taken together with the genomic variation, geographical distribution and differences in environmental habitats [18], [21], [23], comparative genomic studies suggest that strains associated with human melioidosis may possess an accessory genome that differs from animal and environmental strains [24]. We hypothesize that differences in virulence may be associated with variations in metabolic and regulatory capabilities among *B. pseudomallei* strains.

In this study we compared three *B. pseudomallei* genomes, from clinical strains MSHR668, K96243 and 1106a, seeking to identify metabolic characteristics that might explain why MSHR668 is more virulent than K96243 and 1106a in a mouse model of *B. pseudomallei* infection. Analyses focused on genomic features, including metabolic, virulence and regulatory genes that were present in MSHR668 but absent from both K96243 and 1106a. Features present in K96243 and 1106a but absent from MSHR668 were noted, and we also identified virulence-associated genes that were present in all three genomes. Here we have identified genomic features that may contribute to variations in virulence noted among *B. pseudomallei* isolates.

## Results

### Comparative Virulence of *B. pseudomallei* Isolates

For the purposes of this manuscript, we measured the LD_50_ upon intraperitoneal (IP) challenge to assess potential differences in virulence among the three *B. pseudomallei* strains. While studies evaluating clinical infection are complicated by a range of factors such as host risk factors, exposure routes and dose of exposure, experimental studies using inbred mice were used in an attempt to limit the number of host factors that may contribute to differences. Experiments involving infections of BALB/c and C57BL/6 mice [25] with *B. pseudomallei* strains K96243, MSHR668, and 1106a revealed differences in LD_50_ values among the *B. pseudomallei* strains. LD_50_ values were calculated after 21 and 60 days post-challenge. Differences were more pronounced in the BALB/c model, where the LD_50_ values of MSHR668 were 30 to 100-fold lower than those of K96243 and 1106a (Table 1). The LD_50_ values for MSHR668 were also lower in the C57BL/6 model, although the differences were not as great. Since K96243 and 1106a had similar virulence properties in both mouse infection models, we were interested in identifying the genomic features that these strains shared but were not common to MSHR668.

**Table 1:** LD_50_ values from intraperitoneal exposure of BALB/c and C57BL/6 mice to *B. pseudomallei* strains MSHR668, K96243 and 1106a.

BALB/c	Strain	Day 21 LD50	95% HPD Credible Interval	Day 60 LD50	95% HPD Credible Interval
	K96243	6.15×10^4^	2.65×10^5^–1.38×10^5^	3.45×10^4^	1.18×10^4^–1.06×10^5^
	668	1.34×10^2^	37 −4.53×10^2^	1.35×10^2^	37–4.5×10^2^
	1106a	4.15×10^4^	1.69×10^4^–9.55×10^5^	4.14×10^4^	1.70×10^4^–9.39×10^5^
**C57BL/6**	**Strain**	**Day 21 LD50**	**95% HPD Credible Interval**	**Day 60 LD50**	**95% HPD Credible Interval**
	K96243	2.24×10^6^	1.15×10^6^–4.29×10^6^	1.09×10^6^	4.97×10^5^–2.25×10^6^
	668	1.70×10^5^	9.93×10^4^–3.01×10^5^	3.18×10^4^	1.34×10^4^–7.24×10^4^
	1106a	3.47×10^6^	1.48×10^6^–8.35×10^6^	1.17×10^6^	4.55×10^5^–3.12×10^6^

HPD: Highest Posterior Density.

### Genome Features

We performed an extensive comparative analysis of the *B. pseudomallei* genomes to identify genomic features that are common and unique among the various strains, and to begin to address differences in virulence and disease presentation. Because the K96243 genome that we downloaded from NCBI contained nearly 1,500 fewer CDS than the other two genomes, we re-annotated all three genomes using the RAST system [26] to ensure consistent comparisons. Table 2 compares the three complete genomes in terms of their general features. Comparisons of the CDS in each genome identified by RAST annotation compared to the original annotations showed that the number of CDS in the K96243 genome increased by 1,317 (18.7%). The numbers of CDS in the MSHR668 and 1106a genomes were also increased, but by smaller percentages (3.5% and 4.2%, respectively). These analyses provided a common annotation platform from which the ensuing comparisons were made.

**Table 2:** General genome features.

Feature	MSHR668	K96243	1106a
Genome size (bp)	7,040,403	7,247,547	7,089,249
No. chromosomes	2	2	2
Genes	6,940	7,116	6,946
Protein coding (RAST annotation)	6,869	7,045	6,875
Protein coding (original annotation)	7,116	5,728	7,174
Mobile elements	72	79	89
rRNA operons	12	12	12
tRNA genes	59	59	59
GC%	68.3	68.1	68.3
Regulatory elements	333	332	328
2-component system	79	81	77

#### Pseudogenes and mobile elements

The number of pseudogenes in each originally annotated genome varied depending on the resource used to identify them. Holden et al. (2004) originally reported that the genome of K96243 contains 26 pseudogenes [19], whereas the IMG system [27] identified 122 pseudogenes in K96243, 5 in MSHR668, and 8 in 1106a. The Pathway Tools [28] identified 136 pseudogenes in K96243, 10 pseudogenes in MSHR668, and 15 pseudogenes in 1106a. Because of this discrepancy, and since we re-annotated the genomes using RAST, which does not include an automatic pseudogene identification step, we identified the potential pseudogenes in each genome using the Psi Phi program [29], which is a comparative method for pseudogene identification. Psi Phi identified no additional pseudogenes in the RAST-predicted CDS of K96243, MSHR668 and 1106a. However, Psi Phi identified a few candidate pseudogenes in the intergenic regions, and there were some CDS with less than full length alignments to known protein sequences in the public databases. Since this report does not focus on pseudogenes, we did not explore these further.

The genomes of K96243 and 1106a contained more genes annotated by RAST [26] as encoding mobile elements (79 and 89, respectively) compared to MSHR668 (S1 Table). The number of genes encoding mobile elements that were identified by RAST annotation of K96243 was greater than the originally reported number of 42 mobile elements in K96243 [19]. This discrepancy is likely due to the higher number of total CDS in the RAST annotation of the K96243 genome.

#### Chromosome alignments

**Figure 1:**
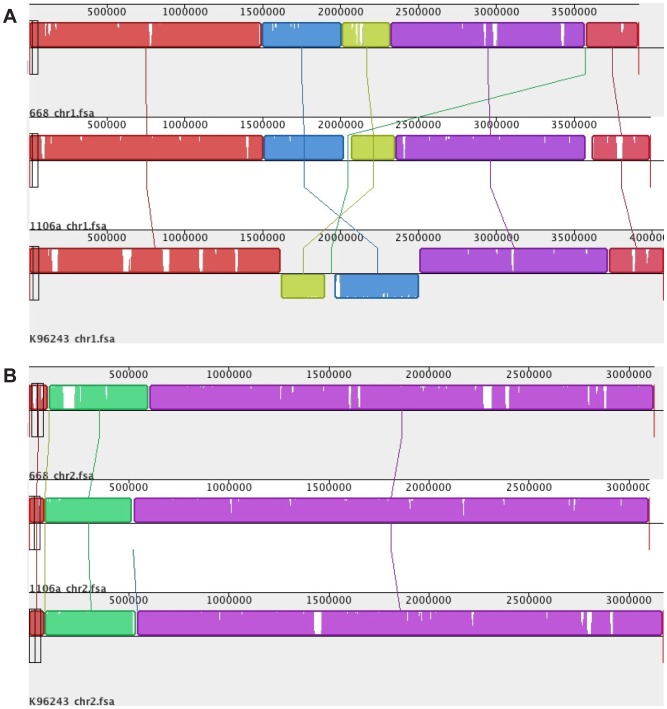
Mauve alignment of *B. pseudomallei* chromosomes 1 (panel A) and 2 (panel B). Homologous regions in the genomes are illustrated as locally collinear blocks of the same color that are linked across the chromosomes. The three genomes showed five homologous regions in chromosome 1, and three homologous blocks in chromosome 2.

Individual chromosomes of *B. pseudomallei* MSHR668, 1106a and K96243 were aligned using Mauve [30], and results showed that they are largely collinear, except for an inversion of the K96243 chromosome 1 and a small gap in between the locally collinear blocks in the inverted region (Fig. 1).

#### Coding sequence comparisons

**Figure 2:**
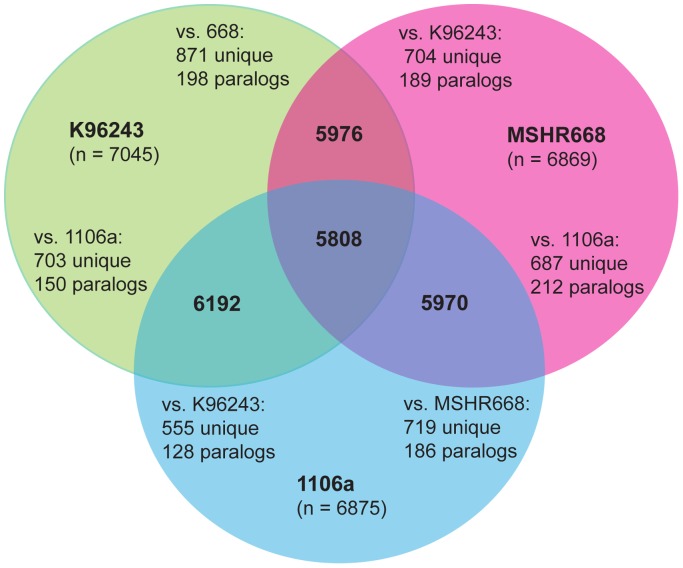
Venn diagram illustrating the numbers of CDS shared by *B. pseudomallei* strains K96243, MSHR668 and 1106a, determined by a bidirectional best BLAST hits analysis. The number of CDS unique to each genome in each pairwise comparison and the number of putative paralogs are shown. The total number of CDS present in each genome is given below the genome name.

The protein coding sequences (CDS) in common among the genomes (putative homologs) were identified by a bidirectional best BLASTp hits analysis. This also enabled the identification of unique genes that were only present in each genome or group of genomes. Fig. 2 shows the results of the analyses for each pair of genomes, as well as all three genomes together. A total of 5,808 CDS were shared by all three genomes. The pairwise comparisons showed 5,976 CDS shared between K96243 and MSHR668, 6,192 CDS in common between K96243 and 1106a, and 5,970 CDS shared between MSHR668 and 1106a.

**Figure 3:**
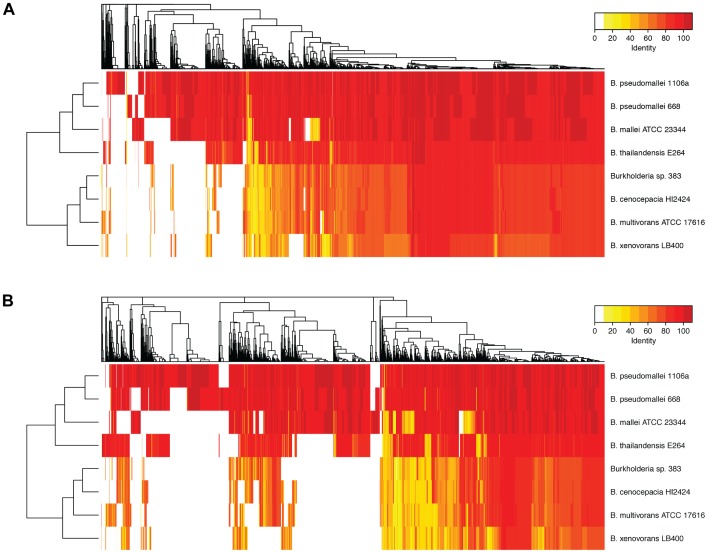
Heatmap displaying best BLAST hits of protein sequences from eight Burkholderia genomes to *B. pseudomallei* K96243 proteins on chromosome 1 (Panel A) and chromosome 2 (panel B). The protein BLAST was run without the filter and an E-value cutoff of 1e-15.

**Figure 4:**
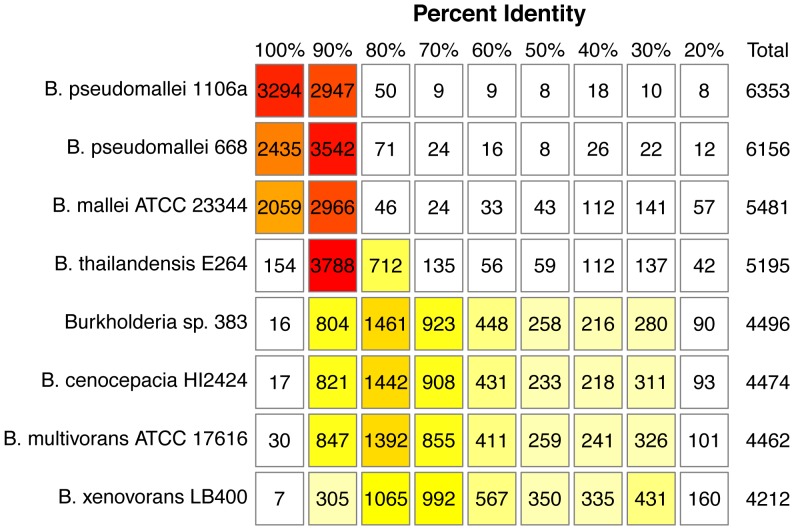
Summary of the number of best BLAST hits matching *B. pseudomallei* K96243 proteins at different percent identity cutoffs.

The distribution of BLASTp hits to strain K96243 is also displayed in a heatmap in Fig. 3. These comparisons included the two *pseudomallei* strains plus *B. thailandensis*, *B. mallei* and other near neighbors to illustrate overall similarities and differences in percent identities across the genomes. The number of best BLASTp hits in these eight *Burkholderia* genomes is also summarized at different percent identity cutoffs in Fig. 4.

#### Gene content comparisons

Although genomic islands (GIs) and their gene content vary greatly among *B. pseudomallei* strains, a thorough comparison of the GIs in the three genomes was already performed [7]. Therefore to investigate potential virulence and metabolism-related genes, we focused on gene clusters and individual CDS (not found in GIs) that were unique to strain MSHR668 and not present in the genomes of 1106a and K96243 (Tables 3 and 4). Many of the genomic features that were present in strain MSHR668 but absent in the genomes of 1106a and K96243 were also present in the genomes of one or more of the other Australian strains, for example strain MSHR305. This result is particularly interesting because of the similar clinical presentations of disease caused by these Australian strains, involving general septicemic infections and the somewhat rare events of encephalomyelitis caused by strains MSHR668 [6] and MSHR305.

**Table 3:** Genes present in the MSHR668 genome that were absent in both K96243 and 1106a.

668 CDS (locus tag)	Function	Present in other *Bp* genomes?
BURPS668_0139	cytidine/deoxycytidylate deaminase	MSHR1043, BDI, BEZ
BURPS668_0798	multidrug ABC transporter permease	1655, S13, MSHR1043, NAU20B-16
BURPS668_0860	CRISPR-associated RAMP Cmr1	no
in RAST annotation (320373.8.peg.1061)	Beta-glucosidase (EC 3.2.1.21)	NCTC 13179, 354e, 1026ab
in RAST annotation (320373.8.peg.1096)	Glycine-rich cell wall structural protein 1.8 precursor	no
BURPS668_1498	phage protein, possible ATP synthase	many
BURPS668_1596	transposase	576, Pakistan 9, 1710ab, MSHR6137
BURPS668_1621	trans-aconitate 2-methyltransferase	no
in RAST annotation (320373.8.peg.1826)	putative HIT domain protein	NCTC 13178, NCTC 13179
BURPS668_2012	gp30	MSHR6137, Pakistan 9, MSHR346, 1710a
BURPS668_2112	Multidrug resistance protein, major facilitator superfamily	NAU20B-16, MSHR511, MSHR146
BURPS668_2138	XRE family transcriptional regulator	no
in RAST annotation (320373.8.peg.2249)	LuxR family transcriptional regulator	576, 1710a, MSHR1043, MSHR6137
BURPS668_2839	putative septum site-determining protein MinD	MSHR1043, 406e, MSHR346
BURPS668_3493	integrase	no
BURPS668_3499	XRE family transcriptional regulator	no
BURPS668_A0076	putative dienelactone hydrolase	1026ab, MSHR346, MSHR338, 406e
BURPS668_A0193	glycosyl transferase group 2 family protein	MSHR6137, MSHR305, NCTC13179, NCTC13178, MSHR511, MSHR146, NAU20B-16
BURPS668_A0194	putative queuine/archaeosine tRNA-ribosyltransferase	NCTC13178, NCTC13179, 1655, MSHR6137
BURPS668_A0197	putative sugar nucleotidyltransferase	MSHR6137, MSHR305, 406e, NCTC13179, NCTC13178, MSHR511, MSHR146, NAU20B-16, 1655
BURPS668_A0198	CDP-glycerol glycerophosphotransferase	MSHR6137, MSHR305, 406e, NCTC13179, NCTC13178, MSHR511, MSHR146, NAU20B-16, 1655, MSHR1043
BURPS668_A0218	flagellar motor switch protein FliM	MSHR305, NCTC 13179, NCTC 13178, MSHR520, MSHR511, MSHR146, NAU20B-16, 406e, 1655
BURPS668_A0222	flagellar hook-basal body protein FliE	same as above
BURPS668_A0227	flagellar protein FliJ	same as above
BURPS668_A0230	signal transduction histidine kinase	same as above
BURPS668_A0231	flagellar hook-length control protein FliK	same as above
BURPS668_A0232	flagellar basal body rod protein	same as above
BURPS668_A0234	flagellar biosynthesis anti-sigma factor	same as above
BURPS668_A0235	flagellar biosynthesis protein FliR	same as above
BURPS668_A0245	flageller rod assembly protein	same as above
BURPS668_A0248	flagellar hook associated protein	same as above
BURPS668_A0249	flagellar hook-length control protein FliK	MSHR305, 406e, 1655
in RAST annotation (320373.8.peg.4106)	membrane protein	no
BURPS668_A0981	integrase	1026ab, NCTC13178, MSHR5858, 576, NAu35A-3, 1710b, others
BURPS668_A1335	DNA-binding protein	MSHR305, MSHR346, MSHR6137, Pasteur 52237, 1710b
BURPS668_A1383	beta-lactamase class A	MSHR305, S13, Pakistan 9, MSHR346, 1710a
BURPS668_A1459	response regulator of the LytR/AlgR family	1710b, MSHR1655, MSHR146, MSHR511, MSHR305, MAU20B-16, MSHR520
BURPS668_A1550	thymidylate kinase	BPC006
BURPS668_A1697	CurM protein	no
BURPS668_A1836	DGPF domain-containing protein	S13, MSHR346, MSHR305, 1710b
BURPS668_A1843	LysR family transcriptional regulator	no
BURPS668_A2058	endoribonuclease L-PSP	MSHR346
BURPS668_A2983	DNA repair ATPase	no

Presence in other *B. pseudomallei* genomes was determined by NCBI BLAST against all genomes in GenBank.

**Table 4:** Genes present in both K96243 and 1106a genomes that were absent in MSHR668.

K96243 and 1106a CDS (locus tag)	Function	Present in other *Bp* genomes?
BPSL0348/BURPS1106A_0385	putative inclusion body protein	MSHR5858, 576, 1026b, 1710b, NCTC13179, others
BPSL0349/BURPS1106A_0386	DNA-directed RNA polymerase subunit beta	MSHR5858, NAU35A-3, MSHR3865, NCTC13179, others
in RAST annotation 272560.34.peg.842/357348.16.peg.782	phage integrase	1258ab, 354ae, MSHR6137, 1026a, MSHR520, MSHR338, MSHR346, MSHR1043
BPSL0763/357348.16.peg.3555	helicase	no
BPSL0764/357348.16.peg.3554	putative restriction enzyme	no
BPSL0765/357348.16.peg.3551	helicase	MSHR5855
272560.34.peg.1128/BURPS1106A_1060	putative OmpA family protein	1106b, BPC006, 576, MSHR6137, 1710b, MSHR1043
BPSL1028/several	transposase	MSHR5858, NCTC13178, 576, 1710b, others
272560.34.peg.1421/BURPS1106A_1350	LysR family transcriptional regulator	NAU35A-3, BPC006, MSHR1153, 1026b, others
BPSL1298/BURPS1106A_1411	histidine kinase	MSHR2243, NCTC13179, MSHR1153, others
BPSL1563/BURPS1106A_2170	membrane protein	MSHR5858, MSHR2243, NAU35A-3, others
BPSL1564/BURPS1106A_2169	Cro/Cl family transcriptional regulator	MSHR5855, MSHR5858, MSHR2243, NAU35A-3, others
272560.34.peg.2810/BURPS1106A_2805	putative periplasmic substrate binding protein	1106b, 576
in RAST annotation 272560.34.peg.3267/357348.16.peg.3156	D-glycero-D-manno-heptose 7-phosphate kinase	S13, MSHR346, MDHR305, BPC006 others
BPSL2817/several	transposase	1026b, MSHR1153, NCTC13179, others
272560.34.peg.3457/BURPS1106A_3460	LysM repeat protein	Pakistan 9, BPC006, 1710a
BPSS0121/BURPS1106A _A0164	beta fimbrial chaperone protein	1026b, MSHR5858, BPC006, others
BPSS0123/BURPS1106A _A0167	beta fimbrial major subunit	1026b, MSHR5858, BPC006, 1710b, others
272560.34.peg.4508/BURPS1106A_A0545	phage holin	MSHR5858, MSHR346, MSHR1655, 1026b, others
BPSS0395/BURPS1106A_A0542	phage protein	MSHR5858, 1710b, MSHR146, others
BPSS0396/BURPS1106A_A0540	phage protein	MSHR305, MSHR520, 576, others
BPSS1075/357348.16.peg.3542	phage tail completion protein	1026b, NCTC13179, others
BPSS1080/357348.16.peg.3545	phage baseplate assembly protein	1026b, NCTC13179, others
BPSS1081/357348.16.peg.3544	phage tail fiber protein	1026b, NCTC13179, others
in RAST annotation 272560.34.peg.6291/357348.16.peg.6156	integrase	1026b, MSHR305, MSHR520, others
BPSS2057/BURPS1106A _A3044	transposase	MSHR1153, NCTC13179, others
in RAST annotation 272560.34.peg.6685/357348.16.peg.6527	transposase	576, MSHR63, MSHR2243, NAU35A-3, others
in RAST annotation 272560.34.peg.6695/357348.16.peg.6539	transposase	1026b, MSHR5855, NCTC13179, others
BPSS2292/BURPS1106A_A3098	universal stress protein	1026b, 1710b, NCTC13179, others
BPSS2298/BURPS1106A _A3104	thioredoxin	1026b, 1710b, 576, others

Presence in other *B. pseudomallei* genomes was determined by NCBI BLAST against all genomes in GenBank.

Addressing differences in the gene content of MSHR668 compared to both K96243 and 1106a, Table 3 lists individual genes (CDS) that were present in the MSHR668 genome but not present in the genomes of both K96243 and 1106a. Table 4 compares the gene content of both K96243 and 1106a, listing CDS that were present in both K96243 and 1106a genomes but absent in MSHR668. Most of the individual genes listed in Table 4 have mobile-element related annotated functions.

#### Metabolic genes and chokepoint reactions

There were some metabolism-related genes in the MSHR668 genome that did not have putative homologs in the K96243 and 1106a genomes (Table 5). The MSHR668 genome had thirteen genes with annotated functions in metabolism that were not present in the K96243 and 1106a genomes. Only four of the functions listed in Table 5 (cytidine/deoxycytidylate deaminase family protein, beta-glucosidase, putative dienelactone hydrolase, beta-lactamase) had additional copies in the MSHR668 genome. Only one gene (BURPS668_1621, encoding trans-aconitate 2-methyltransferase) was associated with a chokepoint reaction by the Pathway Tools [28]. This enzyme transfers one-carbon groups in the reaction that produces S-adenosyl-L-homocysteine from S-adenosyl-L-methionine [31].

**Table 5:** Metabolic and regulatory genes in the MSHR668 genome that were not present in either K96243 or 1106a.

668 Gene	Function	MetaCyc Pathways	KEGG Pathways
Metabolic			
BURPS668_0139*	cytidine/deoxycytidylate deaminase family protein (EC 3.5.4.5/EC 3.5.4.12)	pyrimidine ribonucleosides degradation I, pyrimidine ribonucleotides salvage, purine and pyrimidine metabolism, pyrimidine ribonucleosides degradation II	pyrimidine metabolism
not in previous annot.* (320373.8.peg.1061)	Beta-glucosidase (EC 3.2.1.21)	various sugars converted to beta-D-glucose	Starch and sucrose metabolism Phenylpropanoid biosynthesis Cyanoamino acid metabolism
BURPS668_1621	trans-aconitate 2-methyltransferase (EC 2.1.1.144) #	Reaction: S-adenosyl-L-methionine+trans-aconitate = S-adenosyl-L-homocysteine+(E)-3-(methoxycarbonyl)pent-2-enedioate	same reaction as MetaCyc
not in previous annot. (320373.8.peg.1826)	putative HIT domain protein (nucleotide hydrolase or transferase)	NA	NA
BURPS668_A0076*	putative dienelactone hydrolase (EC 3.1.1.45)	Reaction: dienelactone+H2O < = >2-maleylacetate+H+	Chlorohexane, chlorobenzene, fluorobenzene, toluene degradation
			
BURPS668_A0193	glycosyl transferase group 2 family protein	NA	Mucin-type O-glycan biosynthesis
BURPS668_A0194	putative queuine/archaeosine tRNA-ribosyltransferase (EC 2.4.2.29)	NA	NA
BURPS668_A0197	putative sugar nucleotidyltransferase	NA	NA
BURPS668_A0198	CDP-glycerol glycerophosphotransferase(EC 2.7.8.12)	Reaction: CDP-glycerol+(glycerophosphate)(n) = Cmp+(glycerophosphate)(n+1).	NA
BURPS668_A1383*	beta-lactamase class A	NA	NA
BURPS668_A1550	thymidylate kinase (EC 2.7.4.9)	Reaction: ATP+dTMP< = > ADP+dTDP	Pyrimidine metabolism
BURPS668_A1697	CurM protein	NA	NA
BURPS668_A2058	endoribonuclease L-PSP	NA	NA
Regulatory			
BURPS668_2138*	XRE family transcriptional regulator	NA	NA
not in previous annot.* (320373.8.peg.2249)	LuxR family transcriptional regulator	NA	NA
BURPS668_3499*	XRE family transcriptional regulator	NA	NA
BURPS668_A0230*	signal transduction histidine kinase	NA	NA
BURPS668_A1459*	response regulator of the LytR/AlgR family	NA	NA
BURPS668_A1843*	LysR family transcriptional regulator	NA	NA

*MSHR668 has one or more additional genes for this function.

#candidate chokepoint.

NA: function too general or no pathway associated.

Metabolic genes of interest in the K96243 and 1106a genomes that were not present in MSHR668 (Table 6) included D-glycero-D-manno-heptose 7-phosphate kinase, which is a candidate chokepoint enzyme, a LysM repeat protein and thioredoxin. The thioredoxin function was encoded by additional copies in both K96243 and 1106a genomes. D-glycero-D-manno-heptose 7-phosphate kinase is involved in the biosynthesis of lipopolysaccharide and is a virulence factor and potential protective antigen for *B. pseudomallei* [32].

**Table 6:** Metabolic and regulatory genes in the K96243 and 1106a genomes that were not present in MSHR668.

K96243/1106a Gene	Function	MetaCyc Pathways	KEGG Pathways
Metabolic			
not in prev. annot. (272560.34.peg.3267)	D-glycero-D-manno-heptose 7-phosphate kinase (EC 2.7.1.167)#	ADP-L-glycero-β-D-manno-heptose biosynthesis	Lipopolysaccharide biosynthesis
not in prev. annot./BURPS1106A_3460	LysM repeat protein (putative peptidoglycan hydrolase)	NA	NA
BPSS2298/BURPS1106A _A3104*	thioredoxin (protein disulfide reductase)	NA	NA
Regulatory			
not in prev. annot./BURPS1106A_1350*	LysR family transcriptional regulator	NA	NA
BPSL1298/BURPS1106A_1411*	histidine kinase	NA	NA
BPSL1564/BURPS1106A_2169	Cro/Cl family transcriptional regulator	NA	NA
BPSS2292/BURPS1106A_A3098*	universal stress protein	NA	NA

* K96243 and 1106a have one or more additional genes for this function.

#candidate chokepoint.

NA: function too general or no pathway associated.

#### Metabolic pathways

Metabolic pathways were identified in the three *B. pseudomallei* genomes by Pathway Tools [28] and compared using Pathway Tools, MetaCyc [31], KEGG [33], BLAST analysis [34] and IMG [27]. Pathways comprising central carbon metabolism and the main inputs and outputs are listed in S2 Table. All three of the genomes had components of the main pathways of central carbon metabolism and genes encoding transporters systems, anapleurotic reactions, and pathways for amino acid biosynthesis. The genomes of all three strains had complete pathways to make the amino acids and vitamins that humans obtain from diet and that more fastidious host-restricted intracellular pathogens, such as *F. tularensis*, do not contain. These included histidine, isoleucine, leucine, lysine, methionine, cysteine, phenylalanine, tyrosine, threonine, tryptophan, valine, folate, biotin, lipoic acid, pantothenate, thiamine, riboflavin and vitamin K2 (menaquinone). All of the genomes had genes encoding the cobalamin adenosyltransferase that converts cobalamin to vitamin B12. None of the genomes had genes encoding the enzymes needed to make vitamin K1 (phylloquinone).

Bacterial gene expression is controlled by transcriptional regulators, such as transcription factors and sigma factors. The functions of these proteins in gene expression regulation were first described in *Escherichia coli* [35] and were previously reviewed in *Pseudomonas aeruginosa* [36]. There were many transcription and sigma factors, response regulators, and DNA-binding proteins identified in the *B. pseudomallei* genomes (Table 2). S3 and S4 Tables list the differences in regulatory gene numbers, while Tables 5 and 6 compare gene content between MSHR668 and K96243/1106a. Nearly all of regulatory functions listed in these tables were present in additional copies in the genomes, although their exact gene targets are not known.

#### Virulence genes and metabolism

Table 7 lists virulence genes compiled from online databases [37]–[39]and literature [19], [40] with annotated metabolic and regulatory functions that were present in all three genomes. At least twenty five of the metabolic genes in Table 7 were identified as potential chokepoints by the Pathway Tools.

**Table 7:** Virulence genes in *B. pseudomallei* K96243, 1106a and MSHR668 genomes with metabolic and regulatory functions.

Gene	Annotated Function	Pathways (KEGG, MetaCyc) or process
	Metabolism	
BPSL0338	non-hemolytic phospholipase C (EC 3.1.4.3)	Inositol phosphate metabolism, Glycerophospholipid metabolism, Ether lipid metabolism
BPSL0374	metallo-beta-lactamase superfamily protein	NA
BPSL0395	cytidylyltransferase	various
BPSL0413	lipoate protein ligase B (EC 2.7.7.63)	Lipoic acid metabolism
BPSL0634	oxidoreductase	various
BPSL0808	peptidase; serine protease (EC 3.4.21.-)	various
BPSL0908	phosphoribosylglycinamide formyltransferase (EC 2.1.2.2)	Purine metabolism, One carbon pool by folate, Biosynthesis of secondary metabolites
BPSL1103	endonuclease III (EC 4.2.99.18)#	various
BPSL1196	acetolactate synthase 3 catalytic subunit (EC 2.2.1.6)#	Branched chain amino acid biosynthesis, Butanoate metabolism, C5-branched dibasic acid metabolism, Pantothenate and CoA biosynthesis, Biosynthesis of secondary metabolites
BPSL1561	metallo-beta-lactamase	Hydrolysis of beta-lactam antibiotics
BPSL1776	L-ornithine 5-monooxygenase MbaA/PvdA (EC 1.13.12.-)	Siderophore biosynthesis
BPSL1777	siderophore-related non-ribosomal peptide synthase MbaI	Siderophore biosynthesis
BPSL1778	siderophore related non-ribosomal peptide synthase MbaJ	Siderophore biosynthesis
BPSL1876	phospholipase; phosphoesterase	various
BPSL2403	non-hemolytic phospholipase C (EC 3.1.4.3)	Inositol phosphate metabolism, Glycerophospholipid metabolism, Ether lipid metabolism
BPSL2433	peptidase; Do family protease; serine protease	various
BPSL2519	phosphoserine aminotransferase (EC 2.6.1.52)#	Glycine, serine and threonine metabolism, Methane metabolism, Vitamin B6 metabolism
BPSL2672	epimerase/dehydratase capsule polysaccharide biosynthesis protein	various
BPSL2673	undecaprenyl phosphate N-acetylglucosaminyltransferase; glycoside hydrolase family protein; UDP-D-N-acetylhexosamine:polyprenol phosphate D–N-acetylhexosamine-1-phosphate transferases (EC 2.7.8.-)	various
BPSL2674	NAD-dependent epimerase/dehydratase	various
BPSL2675	glycosyl transferase	various
BPSL2676	glycosyl transferase	various
BPSL2677	O-antigen methyl transferase (EC 2.4.1.-)	various
BPSL2678	glycosyl transferase	various
BPSL2679	NAD-epimerase/dehydratase	various
BPSL2680	O-antigen acetylase WbiA (EC 2.3.1.-)	various
BPSL2683	dTDP-4-dehydrorhamnose reductase (EC 1.1.1.133)#	Biosynthesis of secondary metabolites
BPSL2684	dTDP-6-deoxy-D-glucose-3,5 epimerase (EC 5.1.3.13)#	Biosynthesis of secondary metabolites
BPSL2685	glucose-1-phosphate thymidylyltransferase (EC 2.7.7.24)#	Biosynthesis of secondary metabolites
BPSL2686	dTDP-glucose 4,6-dehydratase (EC 4.2.1.46)#	Biosynthesis of secondary metabolites
BPSL2687	diadenosine tetraphosphatase (EC 3.6.1.41)#	Purine metabolism
BPSL2688	1-acyl-SN-glycerol-3-phosphate acyltransferase; Lysophospholipid Acyltransferases (LPLATs) of Glycerophospholipid Biosynthesis (EC 2.3.1.51)#	various
BPSL2786	acetyltransferase	various
BPSL2787	acyl-CoA transferase; 8-amino-7-oxononanoate synthase (EC 2.3.1.47)#	Biotin metabolism
BPSL2788	UDP-3-O-[3-hydroxymyristoyl] N-acetylglucosamine deacetylase (EC 3.5.1.108)#	various
BPSL2789	capsular polysaccharide biosynthesis fatty acid synthase; type I polyketide synthase WcbR	various
BPSL2790	capsular polysaccharide biosynthesis transmembrane protein; sulfatase (EC 3.1.6.-)	various
BPSL2791	capsular polysaccharide biosynthesis dehydrogenase/reductase; short chain dehydrogenase/reductase family oxidoreductase	various
BPSL2792	capsule polysaccharide biosynthesis/export protein KpsS	various
BPSL2793	D-glycero-d-manno-heptose 1,7-bisphosphate phosphatase (EC 3.1.3.82)#	various
BPSL2794	D-glycero-d-manno-heptose 1-phosphate guanosyltransferase (EC 2.7.7.71)	various
BPSL2795	phosphoheptose isomerase (EC 5.3.1.28)#	Lipopolysaccharide biosynthesis
BPSL2796	sugar kinase; D-glycero-D-manno-heptose 7-phosphate kinase; related to galactokinase and mevalonate kinase (EC 2.7.7.70)#	Lipopolysaccharide biosynthesis
BPSL2797	GDP sugar epimerase/dehydratase; GDP-6-deoxy-D-lyxo-4-hexulose reductase (EC 1.1.1.281)#	Fructose and mannose metabolism, Amino sugar and nucleotide sugar metabolism
BPSL2798	capsular polysaccharide biosynthesis protein; NAD-dependent epimerase/dehydratase	various
BPSL2799	capsular polysaccharide biosynthesis protein	various
BPSL2800	glycosyl transferase	various
BPSL2801	capsular polysaccharide biosynthesis protein	various
BPSL2802	capsular polysaccharide biosynthesis protein	various
BPSL2803	glycosyltransferase	various
BPSL2808	capsular polysaccharide glycosyltransferase biosynthesis protein	various
BPSL2810	GDP-mannose pyrophosphorylase; mannose-1-phosphate guanylyltransferase (EC 2.7.7.13/EC 2.7.7.22)	Fructose and mannose metabolism, Amino sugar and nucleotide sugar metabolism, Biosynthesis of secondary metabolites
BPSL2818	phosphoribosylaminoimidazole synthetase (EC 6.3.3.1)#	Purine metabolism, Biosynthesis of secondary metabolites
BPSL2825	hypothetical protein BPSL2825; para-aminobenzoate synthase, component I PabB (EC 2.6.1.85)#	tetrahydrofolate biosynthesis and salvage, superpathway of chorismate metabolism, superpathway of tetrahydrofolate biosynthesis, 4-aminobenzoate biosynthesis
BPSL3051	anthranilate synthase component II (EC 4 1.3.27)	Phenylalanine, tyrosine and tryptophan biosynthesis, Biosynthesis of secondary metabolites
BPSL3133	imidazole glycerol phosphate synthase subunit HisF (EC 4.1.3.−/EC 2.4.2.-)#	Histidine biosynthesis, Purine biosynthesis
BPSL3168	3-dehydroquinate synthase (EC 4.2.3.4)#	Phenylalanine, tyrosine and tryptophan biosynthesis, Biosynthesis of secondary metabolites
BPSS0067	non-hemolytic phospholipase C (EC 3.1.4.3)	Inositol phosphate metabolism, Glycerophospholipid metabolism, Ether lipid metabolism
BPSS0419	glucose-1-phosphate cytidylyltransferase (EC 2.7.7.33)#	Starch and sucrose metabolism, Amino sugar and nucleotide sugar metabolism, Biosynthesis of secondary metabolites
BPSS0420	CDP-glucose 4,6-dehydratase (EC 4.2.1.45)#	Amino sugar and nucleotide sugar metabolism, Biosynthesis of secondary metabolites
BPSS0421	lipopolysaccharide biosynthesis protein rfbH	Lipopolysaccharide biosynthesis
BPSS0422	aminotransferase	various
BPSS0424	glycosyl transferase group 2	various
BPSS0425	heptosyltransferase (O-antigen related)	Lipopolysaccharide biosynthesis
BPSS0426	heptosyltransferase (O-antigen related)	Lipopolysaccharide biosynthesis
BPSS0427	O-acetyl transferase; galactoside O-acetyltransferase	Lipopolysaccharide biosynthesis
BPSS0428	glycosyl transferase (O-antigen related)	Lipopolysaccharide biosynthesis
BPSS0581	salicylate biosynthesis isochorismate synthase (EC 5.4.4.2)#	Ubiquinone biosynthesis, Biosynthesis of siderophore group nonribosomal peptides, Biosynthesis of secondary metabolites
BPSS0582	isochorismate-pyruvate lyase (EC 4.2.99.21)	Siderophore biosynthesis
BPSS0583	pyochelin biosynthetic protein PchC (EC 3.1.2.-)	Siderophore biosynthesis
BPSS0584	salicyl-AMP ligase; 2,3-dihydroxybenzoate-AMP ligase (EC 2.7.7.58)#	Siderophore biosynthesis
BPSS0586	pyochelin synthetase	Siderophore biosynthesis
BPSS0587	pyochelin synthetase	Siderophore biosynthesis
BPSS0588	pyochelin biosynthetic protein	Siderophore biosynthesis
BPSS0666	peptidase; collagenase (EC 3.4.24.3)	Digestion of native collagen
BPSS0885	N-acylhomoserine lactone synthase; autoinducer synthase BpsI (EC 2.3.1.184)	Quorum sensing
BPSS0946	beta-lactamase precursor	Hydrolysis of beta-lactam antibiotics
BPSS1180	N-acylhomoserine lactone synthase; autoinducer synthetase	Quorum sensing
BPSS1570	N-acylhomoserine lactone synthase; autoinducer synthetase BpmI	Quorum sensing
BPSS1705	3-isopropylmalate dehydrogenase (EC 1.1.1.85)#	Branched chain amino acid biosynthesis, Butanoate metabolism, C5-branched dibasic acid metabolism, Biosynthesis of secondary metabolites
BPSS1825	glycosyltransferase	various
BPSS1826	glycosyltransferase	various
BPSS1828	glycosyltransferase group 1 protein	various
BPSS1829	glycosyltransferase	various
BPSS1830	exopolysaccharide capsular polysaccharide biosynthesis-like tyrosine-protein kinase	capsule biosynthesis
BPSS1831	exopolysaccharide (EPS) capsular polysaccharide biosynthesis related polysaccharide lipoprotein	capsule biosynthesis
BPSS1832	exopolysaccharide (EPS) capsular polysaccharide biosynthesis-like; low molecular weight protein-tyrosine-phosphatase	capsule biosynthesis
BPSS1833	UDP-glucose 6-dehydrogenase 2 (EC 1.1.1.22)#	Pentose and glucuronate interconversions, Ascorbate and aldarate metabolism, Starch and sucrose metabolism, Amino sugar and nucleotide sugar metabolism,Biosynthesis of secondary metabolites
BPSS1834	lipopolysaccharide biosynthesis-like protein; undecaprenyl-phosphate glucose phosphotransferase (EC 2.7.8.31)	NA
BPSS1915	metallo-beta-lactamase	NA
BPSS1993	serine metalloprotease precursor	NA
BPSS1997	class D beta-lactamase	Hydrolysis of beta-lactam antibiotics
	Regulation	NA
BPSL0812	TetR family regulatory protein; multidrug efflux pump repressor protein BpeR	NA
BPSS0887	N-acylhomoserine lactone dependent regulatory protein; autoinducer-binding transcriptional regulator BpsR	NA
BPSS1176	N-acyl-homoserine lactone dependent regulatory protein; ATP-dependent transcriptional regulator LuxR	NA
BPSS1569	N-acylhomoserine lactone-dependent regulatory protein; autoinducer-binding transcriptional regulator BmpR	NA
BPSL1787	extracytoplasmic-function sigma-70 factor	NA
BPSL1805	TetR family regulatory protein; multidrug efflux operon transciptional regulator AmrR	NA
BPSL2347	LuxR family transcriptional regulator	NA
		
BPSL2434	sigma E factor regulatory protein	NA
BPSL2435	sigma E factor negative regulatory protein, RseA family	NA
BPSL2866	oxidative stress regulatory protein OxyR; LysR family transcriptional regulator	NA
BPSS0312	LuxR family transcriptional regulator	NA
BPSS0585	AraC family transcriptional regulator PchR	NA
BPSS1391	AraC family transcriptional regulator	NA
BPSS1520	AraC family transcriptional regulator	NA
BPSS1522	two-component response regulator; LuxR family DNA-binding response regulator	NA

While K96243 GenBank locus tags are listed, genes are present in all three genomes.

NA: no pathway associated with the enzyme.

Various: enzyme may participate in multiple pathways or annotation too general to identify pathways by EC number.

# Candidate chokepoint.

All of the genes in this table were present in various other *B. pseudomallei* genomes, as determined by NCBI BLAST against all genomes in GenBank.

## Discussion

Experimental infection of mouse models with the three *B. pseudomallei* strains showed that the K96243 and 1106a strains from Thailand had similar LD_50_ values in both BALB/c (more susceptible) and C57BL/6 (more resistant) murine infection models, while the Australian strain MSHR668 was more virulent as measured by LD_50_. Given the incredible amount of genomic diversity among *B. pseudomallei* strains, we sought to identify candidate genomic differences that may correlate with variations in virulence. We conducted whole genome comparisons focusing on virulence, metabolism and regulation and identified genes in common among all three genomes. We also identified genes that were present in MSHR668 but absent in K96243 and 1106a (and vice versa). Our findings and the implications on our understanding of melioidosis as a disease are discussed below.

Comparison of the three *B. pseudomallei* genomes revealed genomic differences that included the previously reported variability in GIs [7], [19], which were likely acquired by horizontal transfer [19], as evidenced by their proximity to transposases, integrases, tRNA genes, and the presence of phage-related genes within the GI. This variability in the GI regions may contribute to virulence potential, particularly because these regions can encode a broad array of functions [20]. The intracellular life cycle and adaptation of a pathogen to the host cell environment depends on the expression of virulence factors, which is controlled by regulatory elements, and may be affected by the metabolic state of the pathogen [41]. The genomes of *B. pseudomallei* MSHR668, K96243, and 1106a contained complete gene sets for the core pathways comprising carbon metabolism. They also contained gene sets encoding transporters and utilization pathways for a wide range of carbon substrates, anapleurotic reactions and fatty acid degradation products (S2 Table), providing many potential targets for metabolic regulation.

An important outcome of the metabolic pathway analysis was identification of chokepoint reactions in the three genomes by the Pathway Tools software [28]. Inhibition of an enzyme that consumes a unique substrate might cause accumulation of the substrate and be potentially toxic to the cell. Conversely, inhibition of an enzyme that produces a unique product might result in starvation for that product, which could cripple essential cell functions. Thus, chokepoint enzymes may be essential to the pathogen and therefore represent potential drug targets. We identified two chokepoint reactions among the lists of genes in Tables 5 and 6, which were differentially present in the three genomes. Among the genes in Table 7, we identified twenty-five candidate chokepoint enzymes in common among the three genomes, involved in a variety of metabolic functions. The complete list of chokepoint reactions, including candidates, totals approximately 1,200−1,300 reactions for each genome (data not shown) and requires additional curation and more extensive comparative analysis to determine which ones are the most promising targets. While our findings indicate that there are only a few metabolic differences among the *B. pseudomallei* genomes, it is becoming increasingly apparent that virulence and metabolism are linked together by complex regulatory interactions occurring between intracellular pathogens and their host cells [41]–[45]. We did find a few differences in regulatory gene content between MSHR668 and the other two genomes, in particular the K96243 and 1106a genomes contained more predicted sigma factor encoding genes than MSHR668 (S4 Table). Also the MSHR668 genome encoded additional transcriptional regulators, specifically two XRE family, two LysR family and one LuxR family, that were not present in the other genomes (Table 3). The genomes of both 1106a and K96243 encoded one LysR family and one Cro/CI family regulator that were not present in the MSHR668 genome (Table 4). These results suggest that differences in regulation may contribute to the differences in virulence observed among these strains. Although further work is needed to test this hypothesis, the observed differences in transcriptional regulatory genes may contribute to the differential virulence observed in this study.

Increasing evidence indicates that virulence gene expression is regulated by nutrients in the environment surrounding *B. pseudomallei* [46]. The expression of pathogen genes involved in transport and utilization of nutrients containing carbon and nitrogen is controlled by transcriptional regulators that are activated by the presence of nutrients [47]–[51]. For example, RpoS is involved in the response of *B. pseudomallei* to carbon starvation, heat shock, osmotic stress and oxidative stress. The expression of metabolic pathway genes involved in central carbon metabolism is controlled by RpoS, and by RpoS and BpsI co-regulation [52]. Therefore, the inter-regulation of stress response and metabolic genes by RpoS and BpsI may play an important role in *B. pseudomallei* survival and virulence [52]. RpoS has been reported to play a role in virulence gene expression in *Salmonella typhimurium* [53], and may also influence host macrophage responses to *B. pseudomallei* infection [54], [55]. The polyamines spermidine and putrescine regulate gene expression at the transcriptional level by affecting regulatory protein binding to DNA. The Fur protein is a positive regulator of peroxidase and iron-containing superoxide dismutase expression, but in response to increased iron concentrations, Fur reduces the transcription of iron-regulated promoters [56].

Several studies have examined transcriptional profiles of *B. pseudomallei* during infection [46], [57]–[61]. Results of these efforts support the idea that some virulence functions leading to infection and disease are linked to pathogen metabolism through regulation of gene expression. In some cases, metabolic enzymes may act as virulence factors through their role in providing nutrients to the pathogen during infection. For instance, phosphoserine aminotransferase, encoded by *serC*, is involved in serine and pyridoxal-5-phosphate synthesis, and may be a virulence factor in *B. pseudomallei*, as it is co-expressed with other virulence genes and auxotrophic mutation attenuates virulence [59]. Several studies have shown that some genes involved in metabolic processes and virulence are upregulated in *B. pseudomallei* during infection, while other metabolic genes are downregulated [46], [57], [58], [60], [61]. In spite of the increasing evidence linking metabolism and virulence, further work is needed to thoroughly characterize the overlapping roles of virulence factors, regulators and metabolic pathways in *B. pseudomallei* pathogenicity. Comparative genomic approaches such as those described here can be a key first step in generating hypotheses with respect to the roles of various bacterial factors in virulence.

Bacterial pathogens have evolved strategies to alter their lifestyle depending on whether they are in their natural environment or infecting a host, shifting resources from normal cell functions to the production of virulence factors, and altering metabolism to take advantage of the nutrients provided by host cells to facilitate survival and growth [62]. This should be especially true for *B. pseudomallei* given its presence and survival in a range of soil samples [63]–[68] and ability to cause severe disease in humans. Our comparison of the genomic features of two *B. pseudomallei* strains from Thailand (K96243 and 1106a) to one strain from Australia (MSHR668) revealed that the genomes are very similar in the repertoires of metabolic and virulence genes that they contain, leading to the conclusion that differential virulence studies on a larger scale are warranted. Detailed experiments will be necessary to characterize the relevance of specific genomic features to *B. pseudomallei* metabolism and virulence, and particular attention should be focused on the regulatory mechanisms influencing gene expression. Continued emphasis in this area will be critical to protection against melioidosis, as a better understanding of what constitutes a fully virulent *Burkholderia* isolate may inform better diagnostic and medical countermeasure strategies. The comparative genomic analysis that we present in this report, combined with more detailed functional analyses of metabolic networks, virulence and regulation, shows promise for examining the effects of *B. pseudomallei* and other intracellular pathogens on host cell metabolism and will lay a foundation for future prediction of the virulence of emerging strains.

## Materials and Methods

### Animal Challenges

Mouse challenges and statistical analyses were performed to establish LD_50_ values for each strain of *B. pseudomallei*. The United States Army of Medical Research Institute of Infectious Diseases is compliant with all federal and Department of Defense regulations pertaining to the use of Select Agents. Cultures were initiated by inoculating Glycerol Tryptone broth (GTB-10g/L tryptone, 5 g/L NaCl, 40 ml/L glycerol) with defrosted freezer stock of *B. pseudomallei*. The cultures were grown at 37°C while shaking at 200 RPM for approximately 8–10 hours in order to harvest cells at late logarithmic phase of growth. Challenge doses were prepared according to OD_620 nm_ values and cultures were plated on sheep blood agar plates to confirm the number of colony forming units per milliliter (CFU/ml). At least 5 dose groups were used and 10 mice were included in each group. BALB/c and C57BL/6 mice were ordered from the National cancer Institute-NCI Frederick and were approximately 7–10 weeks of age at time of challenge. Mice were challenged intraperitoneally with bacterial doses suspended in 200 µl of GTB. Mice were observed at least daily for signs of illness or distress and monitoring frequency increased as indicated by the advancement of clinical signs. Challenged mice were observed at least twice daily for 60 days for clinical signs of illness. Humane endpoints were used during all studies, and mice were humanely euthanized when moribund according to an endpoint score sheet. Animals were scored on a scale of 0–12∶0–2 = no clinical signs; 3–7 = clinical symptoms; increase monitoring; greater than or equal to 8 = distress; euthanize. Those animals receiving a score of 8 or greater were humanely euthanized by CO_2_ exposure using compressed CO_2_ gas followed by cervical dislocation. However, even with multiple checks per day, some animals died as a direct result of the infection. Animal research at The United States Army of Medical Research Institute of Infectious Diseases was conducted and approved under an Institutional Animal Care and Use Committee in compliance with the Animal Welfare Act, PHS Policy, and other Federal statutes and regulations relating to animals and experiments involving animals. The facility where this research was conducted is accredited by the Association for Assessment and Accreditation of Laboratory Animal Care, International and adheres to principles stated in the Guide for the Care and Use of Laboratory Animals, National Research Council, 2011.

A Bayesian probit analysis was performed for each *Burkholderia* strain to estimate the lethal dose response curve. Prior distributions for each parameter were assumed to be independent, weakly informative Cauchy distributions with center 0 and scale 10. Using samples from the posterior distributions of the slope and intercept parameters from the probit analysis, the median and 95% credible intervals of the range of dose responses are estimated. Direct comparisons of the posterior samples of the LD50s of each strain permit us to make probabilistic statements about how likely it is that one strain is more or less potent than any other strain, given the observed data.

### Genome Analysis

Whole genome sequences were obtained from NCBI (accession numbers NC_006351.1, NC_006350.1, NC_009074.1, NC_009075.1, NC_009076.1, NC_009078.1). To facilitate consistency in genome comparisons, genomes were annotated with RAST [26]. The GenBank format files for the RAST-annotated genomes are included in S1–S3 Files. The numbers of pseudogenes in each genome were obtained through the software package Psi Phi, which was kindly provided by Prof. Lerat [29]. In preparation for running Psi Phi, annotated protein sequences from each query genome were obtained from NCBI and used to query the nucleotide sequences of the other target genomes using tblastn. To identify potential pseudogenes, the Psi Phi software compares protein sequence matches in a query genome to the GenBank file of the target genome. We identified matches having a blast score with E-value <10^−10^ and a minimal percentage of protein identity of 80% Matches with 80% to 100% protein sequence identity to the query protein were retained. If a query sequence had two matches in close proximity in the target genome (as might result from frameshifts or insertion), the matches were merged if they were <300 nt apart [69].

Mobile genetic elements, transcription factors, sigma factors, response regulators, DNA binding proteins and two-component signal transduction systems were identified in each genome by searching the annotated genomes in the SEED [70]. Functional analysis was accomplished through the RegPrecise database [71].

Whole genome alignments were performed with Mauve [30]. To identify putative homologs among the genomes of *B. pseudomallei* strains K96243, 668 and 1106a, we performed a bidirectional best hits analysis, using BLASTp with an E-value cutoff of 1e^−5^ to obtain liberal best hits for the proteins of each genome compared to the others. Genes x and y from genomes 1 and 2 are considered as homologs if y is the best BLASTp hit for x and vice versa. We used the blast2gi program from the Seals package [72] to format the BLAST results in tabular form. Each pair of genomes was subjected to this analysis. To obtain the CDS shared by all three genomes, the sequences in common to each pair of genomes were compared to generate a list of CDS present in all three genomes. Sequences unique to each genome were identified by comparison of the total number of CDS in each genome to the common sequences from each pairwise comparison. We gathered the sequences that were unique to MSHR668 and not found in either K96243 and 1106a, and those that were unique to both K96243 and 1106a but not found in MSHR668. These sets of sequences were compared to the originally annotated genomes from GenBank, to determine whether RAST annotation predicted similar CDS to the previously annotated genomes in GenBank. Predicted CDS were not included in the unique set if there were high identity hits (>95%) in the original annotation. The locus_tags in Tables 3–5 refer to CDS present in both the RAST and original annotations. To create heatmaps comparing CDS from each *B. pseudomallei* genome to other Burkholderias, we used protein BLAST version 2.2.26+ to compare *B. pseudomallei* K96243 protein translations against eight other Burkholderia proteomes that we also annotated using RAST. We disabled filtering and set the E-value cutoff to 1e^−15^ and then saved the best hit to each subject protein. The best hits were binned into groups based on percent identity (100%, 90–99.9%, 80–89.9%, etc) and then displayed as a heatmap (Fig. 3), which was created in R using complete linkage hierarchical clustering with euclidean distances. A matrix showing the numbers of CDS shared in each pairwise comparison and percent identity was created by counting the number of best hits in each bin (Fig. 4).

Virulence gene lists were compiled from [19], [37]–[40], [73]. Blast analysis was used to compare the virulence gene sequences among the three genomes and between the original and RAST annotations. Metabolic pathways of the original and RAST-annotated *B. pseudomallei* genomes were analyzed using the Pathway Tools version 18.0 [28]. Chokepoint reactions were identified in *B. pseudomallei* MSHR668, K96243 and 1106a using the chokepoint reaction finder, with human reactions excluded.

### Ethics Statement

Animal research at The United States Army Medical Research Institute of Infectious Diseases (USAMRIID) was conducted and approved under an Institutional Animal Care and Use Committee in compliance with the Animal Welfare Act, PHS Policy, and other Federal statutes and regulations relating to animals and experiments involving animals. The facility where this research was conducted is accredited by the Association for Assessment and Accreditation of Laboratory Animal Care, International and adheres to principles stated in the Guide for the Care and Use of Laboratory Animals, National Research Council, 2011. The USAMRIID IACUC approved this animal care and use protocol. USAMRIID policy does not allow approved animal protocol numbers to be published.

## Supporting Information

S1 Table Number of mobile element genes in *B. pseudomallei* genomes.
(DOCX)

S2 Table Metabolic Pathway Comparison among *B. pseudomallei* genomes.
(DOCX)

S3 Table Transcriptional regulatory genes in *B. pseudomallei* genomes.
(DOCX)

S4 Table Sigma factor genes in *B. pseudomallei* genomes.
(DOCX)

S1 File RAST-annotated MSHR668 genome in GenBank file format.
(ZIP)

S2 File RAST-annotated 1106a genome in GenBank file format.
(ZIP)

S3 File RAST-annotated K96243 genome in GenBank file format.
(ZIP)
