# Correction: Interrogation of the *Burkholderia pseudomallei* Genome to Address Differential Virulence among Isolates

**DOI:** 10.1371/journal.pone.0122178

**Published:** 2015-03-25

**Authors:** The PLOS ONE Staff

There is an error in the confidence values listed in the first row. The exponents should be listed as 10^4^ and not 10^5^.

**Table 1:** LD_50_ values from intraperitoneal exposure of BALB/c and C57BL/6 mice to *B. pseudomallei* strains MSHR668, K96243 and 1106a.

BALB/c	Strain	Day 21 LD50	95% HPD Credible Interval	Day 60 LD50	95% HPD Credible Interval
	K96243	6.15x10^4^	2.65x10^5^-1.38x10^5^	3.45x10^4^	1.18x104-1.06x10^5^
	668	1.34x10^2^	37 - 4.53x10^2^	37 - 4.53x10^2^	37 - 4.5x10^2^
	1106a	4.15x10^4^	1.69x10^4^-9.55x10^4^	4.14x10^4^	1.70x10^4^-9.39x10^4^

C57BL/6	Strain	Day 21 LD50	95% HPD Credible Interval	Day 60 LD50	95% HPD Credible Interval
	K96243	2.24x10^6^	1.15x10^6^-4.29x10^6^	1.09x10^6^	4.97x10^5^-2.25x10^6^
	668	1.70x10^5^	9.93x10^4^-3.01x10^5^	3.18x10^4^	1.34x10^4^-7.24x10^4^
	1106a	3.47x10^6^	1.48x10^6^-8.35x10^6^	1.17x10^6^	4.55x10^5^-3.12x10^6^

HPD: Highest Posterior Density

## References

[1] Challacombe JF, Stubben CJ, Klimko CP, Welkos SL, Kern SJ, Bozue J. et al. (2014) Interrogation of the *Burkholderia pseudomallei* Genome to Address Differential Virulence among Isolates. PLoS ONE 9(12): e115951 doi: 10.1371/journal.pone.0115951 25536074 10.1371/journal.pone.0115951 PMC4275268

