# Supplementary material for: Interrogation of the Burkholderia pseudomallei Genome to Address Differential Virulence among Isolates
Source: PLoS One. 2014 Dec 23;9(12):e115951. doi: 10.1371/journal.pone.0115951 (PMC4275268; doi:10.1371/journal.pone.0115951)
Supplement: S1 Table — Number of mobile element genes in B. pseudomallei genomes. (DOCX) [file pone.0115951.s001.docx]

Table S1. Number of mobile element genes in *B. pseudomallei* genomes

| Genome | MSHR668 | K96243 | 1106a |
| --- | --- | --- | --- |
| Mobile elements |  |  |  |
| integrase | 10 | 14 | 10 |
| transposase | 7 | 9 | 10 |
| mobile element protein | 53 | 54 | 67 |
| OrfA | 2 | 2 | 2 |
| Total | 72 | 79 | 89 |
|  |  |  |  |

Data from RAST annotated genomes
