# Supplementary material for: Interrogation of the Burkholderia pseudomallei Genome to Address Differential Virulence among Isolates
Source: PLoS One. 2014 Dec 23;9(12):e115951. doi: 10.1371/journal.pone.0115951 (PMC4275268; doi:10.1371/journal.pone.0115951)
Supplement: S2 Table — Metabolic Pathway Comparison among B. pseudomallei genomes. (DOCX) [file pone.0115951.s002.docx]

Table S2. Metabolic Pathway Comparison among *B. pseudomallei* genomes

| **Metabolic pathway** | **MSHR668** | **K96243** | **1106a** | ***B. mallei* 10229** | ***B.mallei* SAVP1** | *F. tularensis* SchuS4 | *F. tularensis* FSC198 |
| --- | --- | --- | --- | --- | --- | --- | --- |
| **Central carbon metabolism** |  |  |  |  |  |  |  |
| Glycolysis (β-D-glucose to pyruvate) | + | + | + | + | + | inc | inc |
| Gluconeogenesis ((*S*)-malate to β-D-glucose 6-phosphate) | inc | inc | inc | inc | inc | + | + |
| Entner-Doudoroff (6-phospho D-gluconate to pyruvate) | + | + | + | + | + | - | - |
| Pentose phosphate | + | + | + | + | inc | inc | inc |
| TCA cycle | + | + | + | + | inc | inc | inc |
| Glyoxalate cycle | + | + | + | + | + | inc | inc |
| Fatty acid degradation | + | + | + | + | + | + | + |
| Pyruvate metabolism |  |  |  |  |  |  |  |
| decarboxylation to acetyl-CoA | + | + | + | + | + | + | + |
| fermentation to acetate | + | + | + | + | inc | + | + |
| fermentation to ethanol | + | + | + | + | + | - | - |
| oxidation to acetate | + | + | + | + | + | - | - |
| **Presursors to amino acids** |  |  |  |  |  |  |  |
| sulfate reduction to hydrogen sulfide | + | + | + | + | + | inc | inc |
| L-homoserine from L-aspartate | + | + | + | + | + | inc | inc |
| L-homocysteine from hydrogen sulfide and L-homoserine | + | + | + | + | + | - | - |
| **Biosynthesis** |  |  |  |  |  |  |  |
| **Amino acids** |  |  |  |  |  |  |  |
| L-alanine from pyruvate + 2-aminoethylphosphonate | + | + | + | + | + | inc | inc |
| L-arginine from L-glutamate | + | + | + | + | + | inc | inc |
| L-asparagine from L-aspartate | + | + | + | + | + | + | + |
| L-aspartate from L-glutamine and oxaloacetate | + | + | + | + | + | + | + |
| L-cysteine from L-serine and L-homocysteine | + | + | + | + | + | + | + |
| L-cysteine from glutathione | + | + | + | + | + | + | + |
| L-glycine from L-serine | + | + | + | + | + | + | + |
| L-glycine from L-threonine | + | + | + | + | + | + | + |
| L-histidine | + | + | + | + | inc | - | - |
| L-isoleucine from L-threonine | + | + | + | + | + | inc | inc |
| L-leucine from L-valine | + | + | + | + | + | inc | inc |
| L-lysine from L-aspartate | + | + | + | + | + | inc | inc |
| L-methionine | + | + | + | + | + | inc | inc |
| L-proline from L-glutamate | + | + | + | + | + | inc | inc |
| L-phenylalanine from chorismate | + | + | + | + | + | inc | inc |
| L-serine from 3-phospho-D-glycerate | + | + | + | inc | inc | + | + |
| L-threonine from L-aspartate  (includes path from L-homoserine) | + | + | + | + | + | inc | inc |
| L-tryptophan  from L-glutamine and chorismate | + | + | + | + | + | + | + |
| L-tyrosine from L-phenylalanine and from chorismate | + | + | + | + | + | inc | inc |
| L-valine from pyruvate | + | + | + | + | + | inc | inc |
| **Vitamins** |  |  |  |  |  |  |  |
| thiamine (B1) | inc | inc | inc | inc | inc | inc | inc |
| thiamine salvage | + | + | + | + | + | + | + |
| riboflavin (B2) | + | + | + | + | + | + | + |
| pyridoxine (B6) | inc | inc | inc | inc | inc | inc | inc |
| B12 from cobalamin | + | + | + | + | + | - | - |
| 4-aminobenzoate (folate precursor) | + | + | + | + | + | inc | inc |
| biotin from 8-amino-7-oxanoate | + | + | + | + | + | + | + |
| folate (folic acid) from 4-aminobenzoate and 6-hydroxymethyl-dihydropterin diphosphate | + | + | + | + | + | + | + |
| tetrahydrofolate salvage | + | + | + | + | + | + | + |
| lipoate (lipoic acid) | + | + | + | + | + | + | + |
| niacin (nicotinate) via  NAD salvage pathway | - | - | - | - | - | - | - |
| pantothenate (pantothenic acid) | + | + | + | + | + | + | inc |
| vitamin K1 (phylloquinone) | - | - | - | - | - | - | - |
| vitamin K2 (menaquinol-8) | + | + | + | + | + | + | + |
| **Polyamines** |  |  |  |  |  |  |  |
| Putrescene biosynthesis from L-arginine | + | + | + | + | + | inc | inc |
| Spermidine biosynthesis from putrescine | + | + | + | + | + | + | + |
| **Osmoprotectant** |  |  |  |  |  |  |  |
| glycine betaine | + | + | + | + | + | - | - |
| **Transporter systems** |  |  |  |  |  |  |  |
| **import to cytoplasm** |  |  |  |  |  |  |  |
| **amino acids, precursors** |  |  |  |  |  |  |  |
| standard amino acids | + | + | + | + | + | + | + |
| branched chain amino acids | + | + | + | + | + | ? | ? |
| L-alanine | + | + | + | + | + | ? | ? |
| L-arginine | + | + | + | + | + | + | + |
| L-aspartate | + | + | + | + | + | + | + |
| L-cysteine | - | - | - | - | - | + | + |
| GABA | - | - | - | - | - | + | + |
| L-glutamate | + | + | + | + | + | + | + |
| L-glutamine | + | + | + | + | + | ? | ? |
| glutathione | + | + | + | + | + | ? | ? |
| L-leucine | + | + | + | ? | ? | ? | ? |
| L-lysine | + | + | + | ? | + | + | + |
| L-histidine | + | + | + | + | + | ? | ? |
| L-isoleucine | + | + | + | ? | ? | ? | ? |
| D-methionine | + | + | + | + | + | + | + |
| L-methionine | + | + | + | + | ? | ? | ? |
| L-proline | + | + | + | + | + | + | + |
| serine |  |  |  |  |  |  |  |
| L-valine | + | + | + | ? | ? | ? | ? |
| taurine | + | + | + | + | ? | ? | ? |
| peptide/opine/nickel | + | + | + | + | + | ? | ? |
| cyclic peptides | + | + | + | + | + | ? | ? |
| dipeptides | + | + | + | + | + | ? | ? |
| oligopeptides | + | + | + | + | + | + | + |
| spermidine/putrescine | + | + | + | + | + | + | + |
| **vitamins** |  |  |  |  |  |  |  |
| cobalamin | + | + | + | + | + | ? | ? |
| thiamine | + | + | + | + | ? | ? | ? |
| **ions/salts** |  |  |  |  |  |  |  |
| bicarbonate | + | + | + | + | + | ? | ? |
| Ca^2+^ | + | + | + | + | + | ? | ? |
| chromate | + | + | + | + | + | ? | ? |
| Cl^-^ | + | + | + | + | + | + | + |
| Co^2+^ | + | + | + | + | + | + | + |
| Cu^2+^ | + | + | + | + | + | ? | ? |
| Fe^2+^ | + | + | + | + | + | + | + |
| Fe^3+^ | + | + | + | + | + | ? | ? |
| Fe^3+^-siderophore (hemin) | + | + | + | + | + | ? | ? |
| Fe^3+-^siderophore | + | + | + | + | + | ? | ? |
| Fe^3+^-hydroxamate | + | + | + | + | + | ? | ? |
| ferric malleobactin | + | + | + | + | + | ? | ? |
| K^+^ | + | + | + | + | + | + | + |
| Mg^2+^ | + | + | + | + | + | + | + |
| malonate | + | + | + | + | + | ? | ? |
| molybdate | + | + | + | + | + | ? | ? |
| Na^+^ | + | + | + | + | + | + | + |
| Na^+^ + a bile acid | + | + | + | + | ? | + | + |
| Ni^2+^ | + | + | + | + | + | ? | ? |
| nitrate | + | + | + | + | + | ? | ? |
| nitrite | + | + | + | + | + | ? | ? |
| Pb^+^ | + | + | + | ? | ? | ? | ? |
| phosphate | + | + | + | + | + | + | + |
| phosphonate | + | + | + | + | + | ? | ? |
| 2-aminoethylphosphonate | + | + | + | + | + | ? | ? |
| sulfate | + | + | + | + | + | + | + |
| thiosulfate | + | + | + | + | + | ? | ? |
| aliphatic sulfonate | + | + | + | + | + | ? | ? |
| sodium:solute symporter | + | + | + | + | + | + | + |
| anion:cation symporter |  |  |  |  |  |  |  |
| cation:proton antiporter |  |  |  |  |  |  |  |
| cation |  |  |  |  |  |  |  |
| **sugar/carbohydrate/sugar alcohols/carboxylates/etc** |  |  |  |  |  |  |  |
| L-arabinose | + | + | + | + | + | ? | ? |
| fructose | + | + | + | + | + | ? | ? |
| beta-D-galactose | + | + | + | + | + | + | + |
| galactoside | + | + | + | + | + | ? | ? |
| D-glucarate | + | + | + | + | + | + | ? |
| D-gluconate | + | + | + | + | + | ? | ? |
| glucose | + | + | + | + | + | + | ? |
| glucoside | + | + | + | + | + | + | ? |
| glycerol | + | + | + | + | + | + | + |
| sn-glycerol-3-phosphate | + | + | + | + | + | ? | ? |
| glycolate/lactate | + | + | + | + | ? | ? | ? |
| mannitol | + | + | + | + | + | ? | ? |
| mannose | + | + | + | + | + | ? | ? |
| N-acetylglucosamine | + | + | + | + | + | + | ? |
| N-acetylneuraminidate | + | + | + | + | + | ? | ? |
| ribose | + | + | + | + | + | ? | ? |
| sorbitol | + | + | + | + | + | ? | ? |
| sorbose | + | + | + | + | + | ? | ? |
| xylose | + | + | + | + | + | ? | ? |
| carbohydrate | + | + | + | + | + | ? | ? |
| exopolysaccharide | + | + | + | ? | ? | ? | ? |
| sugar | + | + | + | + | + | + | + |
| **purines/pyrimidines/etc** |  |  |  |  |  |  |  |
| cytosine | + | + | + | + | ? | ? | ? |
| nucleoside | + | + | + | + | + | + | + |
| purine | + | + | + | + | ? | ? | ? |
| uracil | + | + | + | + | ? | ? | ? |
| xanthine |  |  |  |  |  |  |  |
| nicotinamide mononucleotide | - | - | - | - | - | + | + |
| **other** |  |  |  |  |  |  |  |
| 2-oxoglutarate | + | + | + | + | + | ? | ? |
| 4-aminobutanoate | + | + | + | + | + | ? | ? |
| 4-hydroxybenzoate | + | + | + | + | + | ? | ? |
| acetate | + | + | + | + | + | + | + |
| benzoate | + | + | + | + | + | ? | ? |
| carnitine | + | + | + | + | + | + | ? |
| choline | + | + | + | + | + | + | ? |
| citrate | + | + | + | + | + | ? | ? |
| C4-dicarboxylate | + | + | + | + | + | + | + |
| dicarboxylate | + | + | + | + | + | ? | ? |
| ethanolamine | + | + | + | + | + | ? | ? |
| glycine betaine | + | + | + | + | + | + | + |
| lipopolysaccharide | + | + | + | + | + | ? | ? |
| lysophospholipid | + | + | + | + | + | ? | ? |
| quaternary amine | + | + | + | + | + | ? | ? |
| toluene | + | + | + | + | + | + | ? |
| **export to periplasmic space** |  |  |  |  |  |  |  |
| L-arabinopyranose (arabinose) | + | - | - | - | - | + | + |
| amino acid | + | + | + | + | + | + | ? |
| beta-D-glucose | + | + | + | + | + | + | ? |
| cation | + | + | + | + | + | + | + |
| Co/Zn/Cd | + | + | + | - | - | - | - |
| K+ | + | + | + | + | + | ? | ? |
| fusaric acid | + | + | + | + | + | ? | ? |
| glutathione ABC transporter | + | + | + | + | + | ? | ? |
| glycine betaine (L-proline antiporter) | + | + | + | + | + | + | + |
| indole-3-acetate | + | + | + | + | ? | ? | ? |
| LipidA | + | + | + | + | + | + | + |
| L-leucine export protein leuE | + | + | + | + | + | ? | ? |
| L-ornithine (antiporter arcD) | + | + | + | + | + | ? | ? |
| Mg^2+^ /Co^2+^ | + | + | + | + | + | ? | ? |
| a polyamine | + | + | + | + | + | ? | ? |
| polysaccharide | + | + | + | + | + | ? | ? |
| capsular polysaccharide | + | + | + | + | + | ? | ? |
| L-threonine | + | + | + | + | + | + | + |
| **Degradation** |  |  |  |  |  |  |  |
| **amino acids** |  |  |  |  |  |  |  |
| L-alanine | + | + | + | - | - | - | - |
| beta-alanine | + | + | + | + | + | - | - |
| L-asparagine | + | + | + | + | + | + | + |
| L-aspartate | + | + | + | + | + | + | + |
| L-arginine | + | + | + | + | + | inc | inc |
| L-cysteine | - | - | - | - | - | - | - |
| L-glutamate | + | + | + | + | + | - | - |
| L-glutamine | + | + | + | + | + | + | + |
| glutathione | + | + | + | + | + | + | + |
| glycine | + | + | + | + | + | + | + |
| L-histidine | + | + | + | + | + | - | - |
| L-isoleucine | + | + | + | + | + | inc | inc |
| L-leucine | + | + | + | inc | inc | - | - |
| L-lysine | - | + | - | - | - | + | + |
| D-methionine | + | + | + | + | + | ? | ? |
| L-methionine | + | + | + | + | + | - | - |
| L-proline | - | - | - | - | - | - | - |
| D-serine | + | + | + | + | + | - | - |
| L-serine | + | + | + | + | + | + | + |
| L-threonine | + | + | + | + | + | + | + |
| L-tryptophan | + | + | + | + | + | - | - |
| L-tyrosine | + | + | + | + | + | inc | inc |
| L-valine | + | + | + | + | inc | inc | inc |
| L-citrulline | + | + | + | + | + | inc | inc |
| L-ornithine | + | + | + | + | + | - | - |
| taurine | + | + | + | + | + | - | - |
| **sugar/carbohydrate/sugar alcohols/carboxylates/etc** |  |  |  |  |  |  |  |
| L-arabinose | - | - | - | - | - | - | - |
| fructose | + | + | + | + | + | + | + |
| beta-D-galactose | inc | inc | inc | inc | inc | inc | inc |
| galactoside  (beta-D-galactoside) | + | + | + | - | - | - | - |
| D-glucarate to 2-phospho-D-glycerate | inc | inc | inc | inc | inc | - | - |
| D-glucarate to alpha-ketpglutarate | inc | inc | inc | inc | inc | inc | inc |
| D-galactarate to alpha-ketpglutarate | + | + | + | + | inc | inc | inc |
| D-gluconate | + | + | + | + | + | - | - |
| glucose (beta-D-glucose) | + | + | + | + | + | + | + |
| glucoside (beta-D-glucoside) | + | + | + | + | + | - | - |
| glycerol and glycerophosphidiester to xylitol | + | + | + | + | + | + | + |
| sn-glycerol-3-phosphate | + | + | + | + | + | + | + |
| glycolate | + | + | + | inc | inc | - | - |
| D-lactate | + | + | + | + | + | + | + |
| L-lactate | + | + | + | + | + | - | - |
| mannitol | + | + | + | + | + | - | - |
| mannose | + | + | + | + | + | + | + |
| N-acetylglucosamine | inc | inc | inc | inc | inc | inc | inc |
| N-acetylneuraminidate | - | - | - | - | - | - | - |
| ribose | inc | inc | inc | inc | inc | - | - |
| sorbitol | inc | inc | inc | inc | inc | inc | inc |
| sorbose | - | - | - | - | - | - | - |
| xylose (alpha-D-xylopyranose) | inc | inc | inc | inc | inc | - | - |
| xylitol | + | + | + | + | + | - | - |
| **polyamines** |  |  |  |  |  |  |  |
| putrescine | + | + | + | - | - | - | - |
| spermidine | + | + | + | - | - | - | - |
| **other** |  |  |  |  |  |  |  |
| allantoin | + | + | + | + | + | - | - |
| thiosulfate to sulfite | + | + | + | + | + | + | + |
| aliphatic sulfonate (isethionate) to sulfite | + | + | + | + | + | - | - |
| 2-oxoglutarate (TCA cycle) | + | + | + | + | + | + | + |
| 4-aminobutanoate | + | + | + | + | - | - | - |
| 4-hydroxybenzoate | + | + | + | + | + | - | - |
| acetate | + | + | + | + | + | ? | ? |
| benzoate | + | + | + | + | + | - | - |
| carnitine | ? | ? | ? | ? | ? | ? | ? |
| choline | + | + | + | + | + | - | - |
| citrate | + | + | + | + | + | - | - |
| C4-dicarboxylate (chorismate) | + | + | + | + | + | + | + |
| dicarboxylate (fumarate, aspartate, succinate) | + | + | + | + | + | + | + |
| ethanolamine | + | + | + | + | + | - | - |
| toluene | ? | ? | ? | ? | ? | ? | ? |
|  |  |  |  |  |  |  |  |

Data obtained from Pathway Tools ([1](#_ENREF_1)), KEGG ([2](#_ENREF_2)) and MetaCyc ([3](#_ENREF_3)). + One or more pathways for utilization, genes representing all pathway steps (enzymes) are present. inc missing 1 or more steps but not all. – Missing all pathway steps. ? missing in annotation, but absence not thoroughly curated for this study.

References

1. **Karp PD, Paley S, Romero P.** 2002. The Pathway Tools software. Bioinformatics **18 Suppl 1:**S225-S232.

2. **Kanehisa M, Goto S.** 2000. KEGG: Kyoto Encyclopedia of Genes and Genomes. Nucleic Acids Res. **28:**27-30.

3. **Caspi R, Altman T, Dale JM, Dreher K, Fulcher CA, Gilham F, Kaipa P, Karthikeyan AS, Kothari A, Krummenacker M, Latendresse M, Mueller LA, Paley S, Popescu L, Pujar A, Shearer AG, Zhang P, Karp PD.** 2010. The MetaCyc database of metabolic pathways and enzymes and the BioCyc collection of pathway/genome databases. Nucleic Acids Res. **38(Database issue):**D473-479.
