# Supplementary material for: Interrogation of the Burkholderia pseudomallei Genome to Address Differential Virulence among Isolates
Source: PLoS One. 2014 Dec 23;9(12):e115951. doi: 10.1371/journal.pone.0115951 (PMC4275268; doi:10.1371/journal.pone.0115951)
Supplement: S3 Table — Transcriptional regulatory genes in B. pseudomallei genomes. (DOCX) [file pone.0115951.s003.docx]

Table S3. Transcriptional regulatory genes in *B. pseudomallei* genomes

| TF family | MSHR668 | K96243 | 1106a | Functions | Reference |
| --- | --- | --- | --- | --- | --- |
| all | 304 | 310 | 306 |  |  |
| AlpA | 0 | 1 | 1 | prophage activation | ([1](#_ENREF_1)) |
| AraC | 36 | 36 | 36 | Arabinose utilization, Iron homeostasis, Rhamnose utilization, Rhamnogalacturonides utilization, drug resistance, metabolite transport, DNA alkylation response | ([2](#_ENREF_2)) |
| ArsR | 3 | 4 | 3 | Arsenic resistance, Cadmium resistance, Cobalt resistance, Zinc resistance, copper resistance, iron homeostasis, heavy metal resistance, toxin-antitoxin system, methionine metabolism, Iron-sulfur cluster biogenesis, sulfur metabolism, Utilization of aromatic compounds | ([2](#_ENREF_2)) |
| AsnC | 8 | 9 | 8 | Metal homeostasis | ([2](#_ENREF_2)) |
| BasR/PmrA | 1 | 1 | 1 | Resistance to cationic antimicrobials | ([3](#_ENREF_3)) |
| BetI (TetR family) | 1 | 1 | 1 | Choline and glycine betaine uptake, Glycine betaine synthesis | ([2](#_ENREF_2), [4](#_ENREF_4)) |
| BkdR (Fis family) | 1 | 1 | 1 | Branched-chain amino acid degradation | ([2](#_ENREF_2)) |
| CatR (LysR family) | 2 | 2 | 2 | Aromatic hydrocarbon utilization | ([4](#_ENREF_4)) |
| Crp | 6 | 6 | 6 | Carbon metabolism, arginine degradation, Carbon monoxide utilization, Uranium reduction, Alkaline stress response, Nitrosative stress response, Nucleoside utilization, Denitrification, Heavy metal resistance, Anaerobic metabolism, Energy metabolism, Nickel homeostasis, Nitrate and nitrite respiration, Nitrogen assimilation, NAD biosynthesis | ([2](#_ENREF_2)) |
| Cro/CI | 0 | 1 | 1 | lambda phage lifecycle | ([5](#_ENREF_5)) |
| CynR | 1 | 1 | 1 | cyn operon | ([6](#_ENREF_6)) |
| CysB (LysR family) | 2 | 2 | 2 | Cysteine biosynthesis | ([4](#_ENREF_4)) |
| DeoR | 5 | 5 | 5 | Glycerol-3-phosphate and mannitol utilization. Deoxyribonucleoside utilization, Fructose utilization, Fucose utilization, Lipopolysaccharide biosynthesis, Glycerol utilization, Inositol utilization, Lactose utilization, Galactose utilization, Formate metabolism, Rhamnose utilization, Rhamnogalacturonides utilization, Sorbitol utilization, Ascorbate utilization, Copper homeostasis, N-acetylgalactosamine utilization | ([2](#_ENREF_2), [4](#_ENREF_4)) |
| FadP (TetR family) | 1 | 1 | 1 | Fatty acid degradation | ([2](#_ENREF_2), [4](#_ENREF_4)) |
| FlhC | 1 | 1 | 1 | Flagellar operon | ([4](#_ENREF_4)) |
| FlhD | 1 | 1 | 1 | Flagellar operon | ([4](#_ENREF_4)) |
| Ferric uptake regulation protein Fur | 1 | 1 | 1 | Iron and zinc homeostasis | ([2](#_ENREF_2)) |
| GabR | 1 | 1 | 1 | Gamma-aminobutyrate utilization | ([2](#_ENREF_2)) |
| GcvA (LysR family) | 3 | 4 | 4 | Glycine metabolism | ([2](#_ENREF_2)) |
| GntR | 19 | 24 | 20 | Gluconate utilization, Galactarate utilization, N-acetylgalactosamine utilization, Alginate utilization, Arabinose utilization, Beta-glucosides utilization, Betaine utilization, Metabolite transport, Amino acid transport, Carbohydrate metabolism, Galactonate utilization, Galacturonate utilization, Fatty acid degradation, Fructoselysine utilization, Fucose utilization, Gamma-aminobutyrate utilization, Glucosamine utilization, Glycolate utilization, Glucomannan utilization, Histidine utilization, Proline and 4-hydrohyproline utilization, Lactate utilization, Mannose utilization, Mannosides utilization, | ([2](#_ENREF_2)) |
| HexR | 1 | 1 | 1 | Central carbohydrate metabolism | ([2](#_ENREF_2)) |
| HrcA | 1 | 1 | 1 | Heat shock response, GroEL GroES,  Heat shock dnaK gene cluster | ([2](#_ENREF_2)) |
| HxlR | 3 | 3 | 3 | Ribulose monophosphate pathway, energy metabolism, Oxidative stress response, Multidrug resistance, Nitrogen metabolism, | ([2](#_ENREF_2)) |
| IclR | 12 | 12 | 13 | Glyoxylate bypass, 3-hydroxybenzoate degradation, pectin and polygalacturonate utilization, sporulation, aromatic compound utilization, Protocatechuate degradation, L-lyxose utilization, allantoin utilization, homogentisate pathway of aromatic compound degradation, lactate utilization D-galatonate catabolism | ([4](#_ENREF_4)) |
| IscR (Rrf2 family) | 1 | 1 | 1 | Alanine biosynthesis,  Iron-sulfur cluster assembly | ([4](#_ENREF_4)) |
| LacI | 1 | 1 | 1 | Carbon catabolism, Central carbohydrate metabolism, Pentose utilization, sugar utilization, Allose utilization, Beta-glucosides utilization, Fructooligosaccharides utilization, Beta-glucosides utilization, Cellobiose utilization, lactose utilization, Galacturonate utilization, Glucuronate utilization, Fructose utilization, Galactose utilization, Galactosides utilization, Fucose-glucose oligosaccharide utilization, Gluconate utilization, Hexulose metabolism, Idonate utilization, Inositol utilization, Pectin utilization, Kojibiose utilization, sucrose utilization, maltose utilization, maltodextrin utilization, mannose utilization, Alpha-galactosides utilization, N-acetylglucosamine utilization, Neotrehalosadiamine biosynthesis, Oligoglucoside utilization, purine metabolism, ribose utilization, Rhamnose utilization, Rhamnose oligosaccharides utilization, Trehalose utilization, Possibly alpha-mannosides utilization, Pyrimidine utilization, Mannuronate utilization, Xylitol utilization | ([2](#_ENREF_2)) |
| LexA | 1 | 1 | 1 | SOS DNA damage stress response, DNA repair | ([4](#_ENREF_4)) |
| LuxR | 12 | 12 | 12 | Competence, cold shock response, Nitrate and nitrite respiration, Protocatechuate degradation, Carbon fixation, Photosynthesis, metabolite transport, trehalose uptake and utilization | ([4](#_ENREF_4)) |
| LysR | 58 | 54 | 54 | Lysine biosynthesis, p-aminobenzoyl-glutamate utilization, Acetoin production, Salicylic acid resistance, energy metabolism, carbon dioxide fixation, citrate metabolism, metabolite transport, cysteine metabolism, sulfite reduction, amino acid transport, glycine metabolism, glutamate metabolism, Tyrosine degradation, Cysteine metabolism, Methionine metabolism, Branched-chain amino acid biosynthesis, lactate utilization, amino acid efflux, methionine biosynthesis, malate utilization, osmotic stress response, nitrogen assimilation, formate metabolism, salt stress response, Aromatic amino acid metabolism, Xanthosine utilization, L-cystine transporter, Cell wall metabolism, glycine and serine utilization | ([2](#_ENREF_2)) |
| MarR | 13 | 13 | 14 | Antibiotic resistance, xinc homeostasis, metabolite transport, fatty acid biosynthesis, Glycine betaine synthesis, 2-Methylhydroquinone and catechol resistance, Peroxide stress response, Flavonoids response, osmotic stress response, Polyamine homeostasis | ([2](#_ENREF_2)) |
| MerR | 9 | 9 | 9 | Multidrug resistance, copper homeostasis, copper resistance, nitrogen assimilation, heat shock response, Branched-chain amino acid degradation, Acyclic terpenes degradation, carbonyl stress response, Superoxide stress response, metal efflux, benzoate degradation, Cobalt-zinc-cadmium resistance | ([2](#_ENREF_2)) |
| MetR | 1 | 1 | 1 | Methionine metabolism; Methionine biosynthesis | ([2](#_ENREF_2)) |
| NrdR | 1 | 1 | 1 | Ribonucleotide reduction; Deoxyribonucleotide biosynthesis | ([4](#_ENREF_4)) |
| NsrR | 2 | 2 | 2 | Nitrosative stress response | ([2](#_ENREF_2)) |
| PadR | 4 | 4 | 4 | Lipoprotein export, Molybdenum homeostasis, Phenolic acid stress response, | ([2](#_ENREF_2)) |
| PchR (AraC family) | 1 | 1 | 1 | Siderophore synthesis regulator | ([4](#_ENREF_4)) |
| PhnF (GntR family) | 2 | 2 | 2 | Alkylphosphonate utilization | ([4](#_ENREF_4)) |
| PhoB (SphR) | 2 | 2 | 2 | Phosphate metabolism, PHO regulon | ([4](#_ENREF_4)) |
| QorR | 1 | 1 | 1 | Energy metabolism | ([2](#_ENREF_2)) |
| RhlR | 1 | 1 | 1 | quorum sensing | ([7](#_ENREF_7)) |
| RpiR | 3 | 3 | 3 | Sugar utilization, Agmatine utilization, Maltose utilization, Gluconate utilization, Central carbohydrate metabolism, N-acetylmuramate utilization, Sialic acid utilization, N-acetylglucosamine utilization, Ribitol utilization, Tagatose utilization, Allose utilization, phosphogluconate repression | ([2](#_ENREF_2)) |
| Rrf2 | 4 | 3 | 2 | Cysteine metabolism, Energy metabolism, Iron-sulfur cluster biogenesis, Nitrosative stress response | ([2](#_ENREF_2)) |
| SolR | 1 | 1 | 1 | Butanol and acetone formation | ([2](#_ENREF_2)) |
| TetR | 23 | 23 | 22 | Multidrug resistance, antibiotic biosynthesis, osmotic stress and toxix chemical response, control of catabolic pathways, differentiation, virulence, Vanillate utilization, transport | ([8](#_ENREF_8)) |
| XRE | 3 | 5 | 3 | Asparagine degradation, proteolysis, Spermidine biosynthesis, Cell wall metabolism, Biofilm formation | ([2](#_ENREF_2)) |
|  |  |  |  |  |  |
| non-specific annotation | 50 | 49 | 53 |  |  |

1. **Kirby JE, Trempy JE, Gottesman S.** 1994. Excision of a P4-like cryptic prophage leads to Alp protease expression in *Escherichia coli*. J. Bacteriol. **176:**2068-2081.

2. **Novichkov PS, Laikova ON, Novichkova ES, Gelfand MS, Arkin AP, Dubchak I, Rodionov DA.** 2010. RegPrecise: a database of curated genomic inferences of transcriptional regulatory interactions in prokaryotes. Nucleic Acids Res. **38:**D111-118.

3. **Tamayo R, Prouty AM, Gunn JS.** 2005. Identification and functional analysis of *Salmonella enterica* serovar Typhimurium PmrA-regulated genes. FEMS Immunol Med Microbiol. **43:**249-258.

4. **Aziz RK, Devoid S, Disz T, Edwards RA, Henry CS, Olsen GJ, Olson R, Overbeek R, Parrello B, Pusch GD, Stevens RL, Vonstein V, Xia F.** 2012. SEED servers: high-performance access to the SEED genomes, annotations, and metabolic models. PLoS One **7:**e48053.

5. **Schubert RA, Dodd IB, Egan JB, Shearwin K, E.** 2007. Cro's role in the CI Cro bistable switch is critical for {lambda}'s transition from lysogeny to lytic development. Genes Dev. .

6. **Sung YC, Fuchs JA.** 1992. The *Escherichia coli* K-12 cyn operon is positively regulated by a member of the lysR family. J Bacteriol. **174:**3645-3650.

7. **Lequette Y, Lee JH, Ledgham F, Lazdunski A, Greenberg EP.** 2006. A distinct QscR regulon in the *Pseudomonas aeruginosa* quorum-sensing circuit. J Bacteriol. **188:**3365-3370.

8. **Ramos J, Martínez-Bueno M, Molina-Henares A, Terán W, Watanabe K, Zhang X, Gallegos M, Brennan R, Tobes R.** 2005. The TetR family of transcriptional repressors. Microbiol Mol Biol Rev **69:**326-356.
