# Supplementary material for: Interrogation of the Burkholderia pseudomallei Genome to Address Differential Virulence among Isolates
Source: PLoS One. 2014 Dec 23;9(12):e115951. doi: 10.1371/journal.pone.0115951 (PMC4275268; doi:10.1371/journal.pone.0115951)
Supplement: S4 Table — Sigma factor genes in B. pseudomallei genomes. (DOCX) [file pone.0115951.s004.docx]

Table S4. Sigma factor genes in *B. pseudomallei* genomes

A. Sigma factors identified in originally annotated genomes by P2TF database (http://www.p2tf.org/)

| Genome | Sigma factor | chromosome 1 | chromosome2 | total |
| --- | --- | --- | --- | --- |
| 668 | all | 11 | 9 | 20 |
|  | Ecf | 7 | 5 | 12 |
|  | RpoD | 3 | 1 | 4 |
|  | RpoN | 1 | 1 | 2 |
|  | Unclassified | 0 | 2 | 2 |
| K96243 | all | 12 | 8 | 20 |
|  | Ecf | 8 | 4 | 12 |
|  | RpoD | 3 | 1 | 4 |
|  | RpoN | 1 | 1 | 2 |
|  | Unclassified | 0 | 2 | 2 |
| 1106a | all | 12 | 7 | 19 |
|  | Ecf | 8 | 4 | 12 |
|  | RpoD | 3 | 1 | 4 |
|  | RpoN | 1 | 1 | 2 |
|  | Unclassified | 0 | 1 | 1 |

B. Sigma factors identified in originally annotated genomes by IMG

| Genome | Sigma factor | chromosome 1 | chromosome2 | total |
| --- | --- | --- | --- | --- |
| 668 | all | 8 | 5 | 13 |
|  | sigma-70 | 3 | 3 | 6 |
|  | RpoD | 1 | 1 | 2 |
|  | RpoE | 1 | 0 | 1 |
|  | RpoH | 1 | 0 | 1 |
|  | RpoS | 1 | 0 | 1 |
|  | flagellar | 1 | 1 | 2 |
| K96243 | all | 6 | 5 | 11 |
|  | sigma-70 | 2 | 3 | 5 |
|  | RpoD | 0 | 1 | 1 |
|  | RpoE | 1 | 0 | 1 |
|  | RpoH | 1 | 0 | 1 |
|  | RpoN | 0 | 1 | 1 |
|  | RpoS | 1 | 0 | 1 |
|  | flagellar | 1 | 0 | 1 |
| 1106a | all | 9 | 5 | 14 |
|  | sigma-70 | 4 | 4 | 8 |
|  | RpoD | 1 | 1 | 2 |
|  | RpoE | 1 | 0 | 1 |
|  | RpoH | 1 | 0 | 1 |
|  | RpoS | 1 | 0 | 1 |
|  | flagellar | 1 | 0 | 1 |

C. Sigma factors identified in RAST annotated genomes

| Genome | Sigma factor | total |
| --- | --- | --- |
| 668 | all | 19 |
|  | sigma-70 | 4 |
|  | RpoD | 2 |
|  | RpoE | 1 |
|  | RpoH | 1 |
|  | RpoN | 4 |
|  | RpoS | 1 |
|  | sigma-24 | 2 |
|  | flagellar | 2 |
|  | PvdS | 1 |
|  | other | 1 |
| K96243 | all | 22 |
|  | sigma-70 | 6 |
|  | RpoD | 2 |
|  | RpoE | 1 |
|  | RpoH | 1 |
|  | RpoN | 6 |
|  | RpoS | 1 |
|  | sigma-24 | 2 |
|  | flagellar | 1 |
|  | PvdS | 1 |
|  | other | 1 |
| 1106a | all | 22 |
|  | sigma-70 | 6 |
|  | RpoD | 2 |
|  | RpoE | 1 |
|  | RpoH | 1 |
|  | RpoN | 6 |
|  | RpoS | 1 |
|  | sigma-24 | 2 |
|  | flagellar | 1 |
|  | PvdS | 1 |
|  | other | 1 |
